# Epigenetic regulation of ACSL4 via H2A monoubiquitylation connects lipid metabolism to BAP1-mediated ferroptosis

**DOI:** 10.1038/s41418-025-01624-2

**Published:** 2025-11-27

**Authors:** Kexin Fan, Shuting Zhou, Yakun Ren, Jingwen Xiong, Hua Wang, Yaxin Fu, Yuhan Chen, Bobo Wang, Kun Fan, Min Gao, Tingli Guo, Xiaofeng Wei, Lianying Jiao, Jiejun Shi, Chenguang Ding, Yilei Zhang

**Affiliations:** 1 Department of Biochemistry and Molecular Biology, School of Basic Medical Sciences, Department of Kidney Transplantation, Department of Organ Procurement and Allocation, The First Affiliated Hospital, Xi’an Jiaotong University, Xi’an, Shaanxi China; 2 Key Laboratory of Spine and Spinal Cord Injury Repair and Regeneration of Ministry of Education, Tongji Hospital affiliated to Tongji University, Frontier Science Center for Stem Cell Research, School of Life Sciences and Technology, Tongji University, Shanghai, China; 3 School of Pharmacy, Xi’an Jiaotong University, Xi’an, Shaanxi China

**Keywords:** Cancer metabolism, Epigenetics

## Abstract

The tumor suppressor BRCA1-associated protein 1 (BAP1) encodes a nuclear deubiquitinase that specifically removes H2A monoubiquitination at Lys119 (H2Aub) and plays a crucial role in the epigenetic regulation of gene expression through cooperating with several transcriptional factors and chromatin-modifying enzymes. Our previous studies have confirmed that BAP1 represses SLC7A11-mediated cystine metabolism and promotes ferroptosis-dependent tumor suppression. However, how BAP1 regulates gene expression at the genome level and whether additional mechanisms are involved in the BAP1 regulation of ferroptosis remain unclear. Here, we integrate multi-omics analyses to explore the effects of BAP1-mediated H2Aub deubiquitination on the regulation of chromatin accessibility and gene transcription. Notably, we identified a novel target gene, ACSL4, which is positively regulated by BAP1 and contributes to BAP1-mediated ferroptosis. Importantly, genetic knockout or pharmacological inhibition of ACSL4 prevents the upregulation of lipid biosynthesis and ferroptotic cell death caused by BAP1. In addition, we demonstrated that BAP1-mediated regulation of gene expression and ferroptosis is dependent on ASXL family members instead of other BAP1-associated factors like FOXK1/2, HCFC1, and OGT. Together, our findings uncover a previously unappreciated epigenetic mechanism underlying the regulation of ACSL4 by H2A monoubiquitination, which connects ACSL4-mediated lipid metabolism to ferroptosis driven by BAP1, providing new insights into the understanding of metabolic regulation of BAP1-related diseases such as cancers.

## Introduction

The BRCA1-associated protein 1 (BAP1) contains a ubiquitin carboxyl-terminal hydrolase domain and exhibits deubiquitinating enzyme (DUB) activity, which mainly functions to remove monoubiquitin from histone 2A at lysine 119 (H2Aub) within the nucleus [1]. As a core component of the polycomb repressive deubiquitinase (PR-DUB) complex, BAP1 interacts with several other proteins, including other BAP1-associated proteins like host cell factor 1 (HCF1; also known as HCFC1), O-linked N-acetylglucosamine transferase (OGT), the forkhead transcription factors FoxK1 and FoxK2 (FOXK1-2), the lysine-specific demethylase 1B (KDM1B), and additional sex combs-like proteins (ASXL1-3) [2, 3]. Through BAP1-mediated epigenetic regulation of gene transcription, the PR-DUB complex modulates various cellular processes and contributes to tissue development [1, 4]. Importantly, BAP1 acts as a tumor suppressor since cancer-associated BAP1 mutations are frequently observed in uveal melanoma, renal cell carcinoma, mesothelioma, and other malignancies [2]. Of note, tumors related to germline pathogenic variants in BAP1 have been recognized as BAP1-associated tumor predisposition syndrome (BAP1-TPDS), and clinical practice guidelines have been developed for the diagnosis and surveillance of BAP1-TPDS, recently launched by the Clinical Guideline Working Group of the CanGene-CanVar project [5], highlighting the need to decipher the mechanistic regulation of tumor suppression by BAP1.

Metabolic reprogramming is a critical hallmark of cancer, enabling tumor cells to adapt to stressful environments and enhance their survival as well as proliferation [6]. This process is primarily distinguished by modifications in cellular metabolism and energy utilization patterns [7]. Transformations across diverse biological pathways and metabolic substances have emerged as crucial factors fueling the growth of cancer cells, encompassing glucose, lipids, amino acids, and vitamins [8–10]. Targeting nutrient metabolism, like depleting extracellular amino acids or inhibiting transporters responsible for importing corresponding nutrients, significantly suppresses tumor growth and even induces regulated cell death, including apoptosis, ferroptosis, and disulfidptosis, which has emerged as a key strategy in cancer treatments [11–14]. Ferroptosis is an iron-dependent form of regulated cell death driven by lipid peroxidation [15]. The subunit of cystine-glutamate antiporter SLC7A11 contributes to ferroptosis resistance through uptaking cystine and promoting glutathione (GSH) biosynthesis, which is utilized by glutathione-dependent peroxidase 4 (GPX4) to detoxify lipid peroxidation [16]. While ACSL4, an isozyme of the long-chain fatty-acid-coenzyme A ligase family, catalyzes the conversion of free long-chain fatty acids (FAs) into fatty acyl-CoA esters, and thereby plays a pivotal role in lipid biosynthesis and ferroptosis induction [17]. Interfering with cystine metabolism and inducing ferroptosis have been proven to effectively suppress tumor growth and significantly improve the efficacy of chemotherapy, radiotherapy, and immunotherapy [18–20]. Further understanding of the metabolic regulation of ferroptosis will provide us with additional opportunities to defeat cancers in the future.

The deubiquitinase activity of BAP1 is known to contribute to multiple cellular processes, including the maintenance of genomic stability, cell cycle regulation, and apoptosis [21]. However, its role in metabolic reprogramming within tumor cells remains poorly defined. Our previous reports found that tumor suppressor BAP1 could inhibit cystine uptake through suppressing SLC7A11 expression in an H2A-deubiquitination-dependent manner, therefore promoting lipid peroxidation-induced ferroptosis and suppressing tumor in vivo [14], revealing the epigenetic regulation of cystine metabolism and ferroptosis in the context of tumor suppression. Nevertheless, the multifaceted mechanisms by which BAP1 exerts its tumor suppressor function, particularly how BAP1 regulates the level of H2Aub on chromatin and whether additional transcriptional targets of BAP1 mediate its cellular functions, are not yet fully elucidated. This study aims to investigate the specific mechanisms of BAP1 regulation of other target genes and relevant biological processes in cancer cells. Multi-omics analyses were employed to explore transcriptional targets and cellular functions of BAP1. Furthermore, we established the epigenetic regulation of lipid metabolism in BAP1-mediated ferroptosis.

## Results

### BAP1 regulates chromatin accessibility and transcription of metabolism-related genes in an H2Aub-dependent manner

**Fig. 1:**
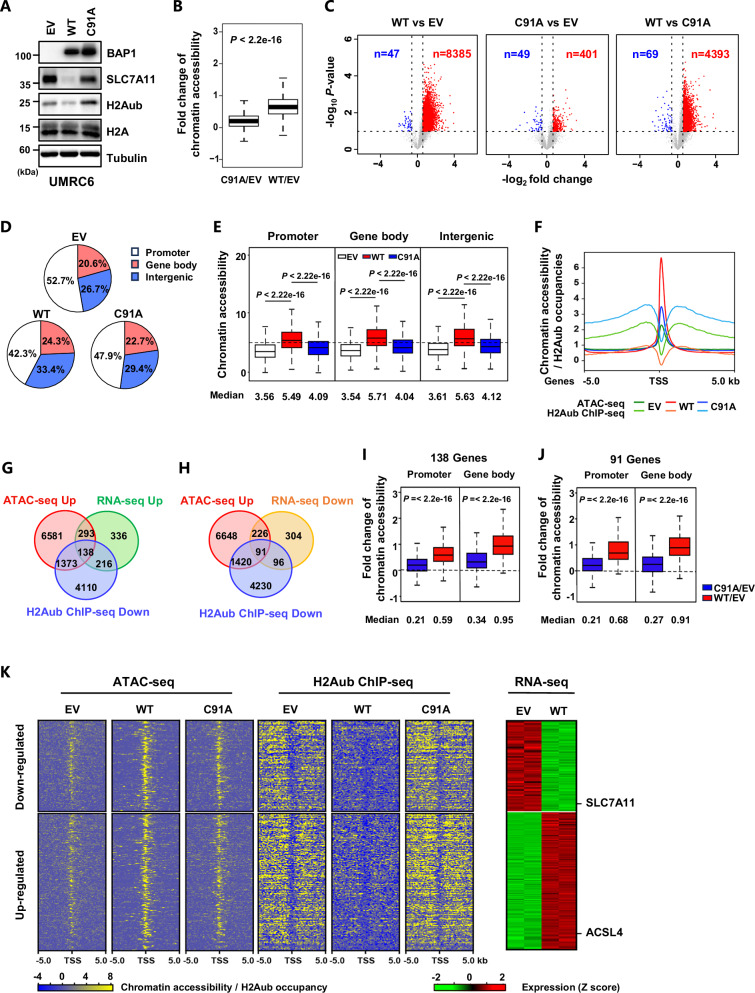
BAP1 regulates target gene transcription and metabolism-related biological processes through chromatin accessibility in an H2Aub-dependent manner. **A** Restoring *BAP1* WT but not C91A in UMRC6 cells decreased H2Aub and its target gene SLC7A11 level. **B** Box plot showing fold changes of chromatin accessibility in BAP1-WT or C91A compared with empty vector (EV) cells. Two-tailed unpaired Student’s *t*-test. **C** Volcano plots of ATAC-seq data for *BAP1* WT or C91A compared with EV cells. The red and blue dots represent genes with at least 1.5-fold increase or decrease of chromatin accessibility in *BAP1* WT (left) or C91A (middle) compared with EV cells. **D** Each pie chart illustrates the distribution of ATAC-seq signal intensities across various genomic regions. The percentages denote the proportion of accessible chromatin regions within each genomic feature category for the respective group. **E** Box plot showing the distribution of chromatin accessibility in BAP1-WT or C91A and empty vector (EV) cells. Two-tailed unpaired Student’s *t*-test was used. **F** Line graph showing chromatin accessibility and H2Aub occupancies around TSS in *BAP1* WT and C91A compared with EV cells. TSS transcriptional start site. **G** Venn diagram showing the overlap among 8385 genes with increased chromatin accessibility, 5837 genes with decreased H2Aub occupancies, and 983 upregulated expressed genes (FC > 1.5, FDR < 0.05) upon restoring BAP1 in UMRC6 cells. **H**. Venn diagram showing the overlap among 8385 genes with increased chromatin accessibility, 5837 genes with decreased H2Aub occupancies, and 717 downregulated expressed genes (FC > 1.5, FDR < 0.05) upon restoring BAP1 in UMRC6 cells. Box plots of log2-fold changes of the chromatin accessibility in promoter and gene body regions for 138 genes (**I**) and 91 genes (**J**). **K** Left three panels: Heatmaps illustrating chromatin accessibility around the TSS of 91 downregulated and 138 upregulated genes. The middle panel displays the H2Aub enrichment profiles near the TSS. The right panel shows the expression levels of the corresponding genes in empty vector (EV) and BAP1 wild-type (WT) cells. These data highlight the epigenetic regulation of gene expression by BAP1, particularly in genes involved in iron and lipid metabolism.

First, we generated empty vector (EV), wild-type BAP1 (WT)- or BAP1-C91A (DUB-inactive mutant)-restored cell lines in BAP1-deficient UMRC6 (containing a frameshift mutation in *BAP1* gene) cells, and the Western blotting assay confirmed that BAP1-WT but not its inactive mutant BAP1-C91A dramatically decreased the levels of H2Aub and SLC7A11 (Fig. 1A), a transcriptional target of BAP1 identified in our previous study [14]. To determine if BAP1 regulates H2Aub occupancies and corresponding transcriptional alterations in the genome through changing chromatin accessibility, we performed ATAC-seq in these cell lines, and found that re-expression of WT rather than its C91A mutant in UMRC6 cells significantly increased global chromatin accessibility (Fig. 1B). To be more specific, the accessibilities of 8385 genes are increased in WT compared to those of EV cells, and only 401 genes’ accessibilities are increased in the BAP1-C91A mutant. Moreover, comparison between WT and C91A revealed increased chromatin openness in a total of 4393 genes (Fig. 1C). Notably, there were fewer than 50 genes with decreased chromatin openness upon re-expression of WT or mutant BAP1 (Fig. 1C), consistent with the model that H2Aub level is positively associated with chromatin compaction [22, 23]. Distribution analysis revealed different levels of chromatin decompaction in EV, WT, and C91A cells, and it indicated that the chromatin accessibilities of the promoter and gene body regions are increased in WT compared to C91A (Fig. 1D, E). Next, we aimed to explore the effect of chromatin accessibility on gene expression regulated by BAP1. Transcriptional changes in EV and WT cells based on our previous RNA-seq data were analyzed [14], and 1700 differentially expressed genes (983 upregulated genes and 717 downregulated genes) upon BAP1 re-expression were obtained (Fig. S1A). Gene ontology (GO) and Kyoto Encyclopedia of Genes and Genomes (KEGG) analysis of BAP1-upregulated and -downregulated genes revealed that BAP1-regulated genes were enriched in diverse cellular processes, particularly metabolism-related biological processes, among which “response to oxidative stress” and “ferroptosis” were included in both (Fig. S1B, C). We further conducted gene set enrichment analysis (GSEA) on the genes with different significance in BAP1-dependent gene expression. GSEA revealed that genes associated with ferroptosis and FA biosynthesis are substantially enriched in gene expression differences between wild-type and BAP1 mutant samples (Fig. S1D, E), indicating the important role of BAP1 in the regulation of ferroptosis. To reveal the relationship between BAP1-mediated H2Aub reduction and the chromatin accessibility in the regulation of gene expression, we utilized previous H2Aub ChIP-seq data for EV, WT, and C91A cell lines (Fig. S1F) [14]. Notably, ATAC-seq peaks profile around transcriptional start sites (TSSs) of BAP1-regulated genes revealed that the genes regulated by BAP1 displayed increased ATAC signals centered on TSSs upon re-expression of BAP1-WT, but not C91A, and were accompanied by a reduction in H2Aub binding (Fig. 1F). Integration of ATAC-seq, RNA-seq and H2Aub ChIP-seq datasets identified 138 and 91 target genes with increased chromatin accessibility and decreased H2Aub levels which were upregulated and downregulated respectively at transcriptional level by BAP1 (Figs. 1G, H and S1G and Supplementary Material). Consistently, the fold changes in chromatin openness of these genes’ promoter or gene body regions were similar to those in the total set of 8385 genes (Fig. 1I, J). Heatmap visualization around TSSs revealed that the genes upregulated or downregulated by BAP1 displayed reduced H2Aub signals and increased ATAC signals centered on TSSs (Fig. 1K). Together, our genome-wide analyses established a model in which BAP1 regulates target gene expression by modulating chromatin accessibility through H2Aub deubiquitination.

### BAP1 promotes ferroptosis through mechanisms that extend beyond SLC7A11-dependent cystine metabolism

**Fig. 2:**
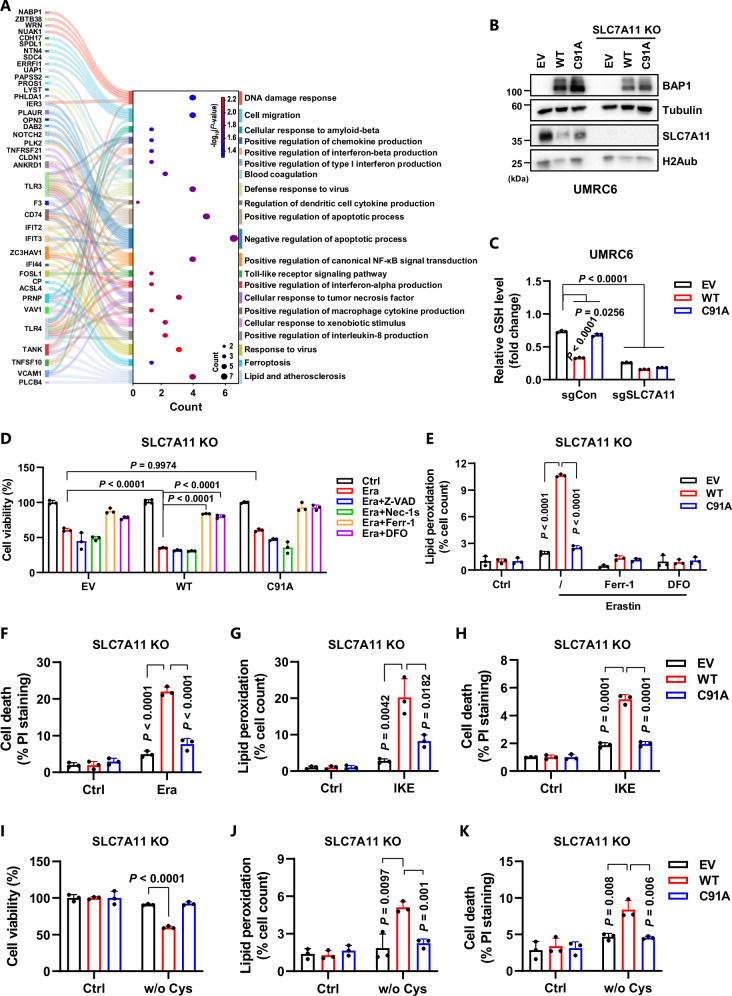
BAP1 promotes ferroptosis through mechanisms that extend beyond SLC7A11-dependent cystine metabolism. **A** The Sankey diagram visualizes that the 138 genes with >1.6-fold H2Aub reduction, >1.5-fold chromatin opening, and >1.5-fold gene expression increased were enriched in the corresponding biological metabolic pathway. **B** Western blotting analysis of BAP1, SLC7A11, and H2Aub levels in UMRC6 cell lines. *n* = 3. **C** Relative GSH levels of indicated UMRC6 cells. **D** Bar graph showing cell viability in indicated cells treated with erastin (10 μM) combined with 5 μM Z-VAD-FMK (Z-VAD), 2 μM Necrostatin-1s (Nec-1s), 2 μM ferrostatin-1 (Ferr-1), or 100 μM deferoxamine (DFO). **E** Lipid peroxidation was assessed by flow cytometry after C11-BODIPY staining in the indicated cells treated with erastin. **F** Cell death was measured in SLC7A11-KO UMRC6 cells restored with *BAP1* WT or C91A mutant and treated with erastin (10 μM) for 24 h. **G** Lipid peroxidation was assessed by flow cytometry after C11-BODIPY staining in the indicated cells treated with IKE. **H** Cell death was measured in SLC7A11-KO UMRC6 cells restored with *BAP1* WT or C91A mutant and treated with IKE (10 μM) for 24 h. **I** Cell viability was measured by CCK-8 assay in SLC7A11-KO-EV, -BAP1, -C91A UMRC6 cells cultured in cystine-containing/-free medium. **J** Lipid peroxidation was assessed by flow cytometry after C11-BODIPY staining in the indicated cells treated with cystine restriction. **K** Cell death was measured in the indicated cell lines and treated with cystine deficiency for 48 h. Error bars are mean ± SD. All *P* values were calculated using a two-tailed unpaired Student’s *t*-test. *n* ≥ 3 independent repeats unless specified. ns not significant (*P* > 0.05).

Next, we sought to explore the relative functions mediated by BAP1-targeted genes identified from the above analysis (Fig. 1K). GO and KEGG analyses of 138 BAP1-upregulated and 91 BAP1-downregulated genes with increased chromatin accessibility and decreased H2Aub levels revealed that they were enriched in multiple metabolism-related biological pathways (Figs. 2A and S2A). It is worth noting that several ferroptosis-related genes were included in the 138 upregulated gene list (Fig. 1K). These newly identified target genes of BAP1 included ceruloplasmin (CP), acyl-CoA synthetase long chain family member 4 (ACSL4), and prion protein gene (PRNP) (Fig.2A), which have been proven to regulate ferroptosis [24–26]. Our previous publication has demonstrated that tumor suppressor BAP1 could promote ferroptosis through inhibiting SLC7A11 expression and cystine uptake in a H2Aub-dependent manner, but whether additional pathways mediate the regulation of ferroptosis by BAP1 remains unclear. To test this hypothesis, we constructed cell lines overexpressing WT or BAP1-C91A in SLC7A11-knockout UMRC6 cells, and confirmed that the H2Aub level was indeed dramatically reduced in WT cells compared to that of EV or C91A cells under SLC7A11-knockout condition (Fig. 2B). In addition, we detected the GSH level in the above cell lines, and observed that BAP1-WT rather than BAP1-C91A could significantly decrease GSH levels in UMRC6 cells, but the effect was largely abolished due to the low basal GSH level in SLC7A11-KO cells (Fig. 2C). BAP1 could promote ferroptosis induced by physiological removal of cystine in the medium or pharmacological inhibitors like erastin and IKE. We found that WT significantly potentiated erastin-induced ferroptosis compared with EV or C91A and increased the levels of lipid peroxidation and cell death, since erastin-induced cell death could be fully suppressed by ferroptosis inhibitors such as ferrostatin-1 (Ferr-1) or the iron chelator deferoxamine (DFO) rather than other cell death inhibitors such as Z-VAD-FMK (apoptosis inhibitor), Nec-1s (necroptosis inhibitor) (Figs. 2D–F and S2B, C). Similarly, IKE treatment led to elevated lipid peroxidation and cell death in WT but not EV or C91A cells under SLC7A11-KO conditions (Figs. 2G, H and S2D, E). Likewise, WT but not its C91A mutant significantly reduced cell viability of SLC7A11-KO UMRC6 cells in the absence of cystine, accompanied by the accumulation of lipid ROS and an increase in cell death (Figs. 2I–K and S2G). Moreover, cystine deprivation suppressed cell viability of SLC7A11-KO cells with wild-type BAP1 in a concentration-dependent manner (Fig. S2F). In addition, we generated an SLC7A11-KO cell line and restored WT or C91A in another *BAP1*-deficient cell line, NCI-H226, followed by IKE or cystine-free medium treatments, and consistent results of WT but not C91A significantly suppressing viability were observed (Fig. S2H–J). These data demonstrated that BAP1 could promote ferroptosis in the absence of SLC7A11.

### ACSL4 is a novel transcriptional target of BAP1

**Fig. 3:**
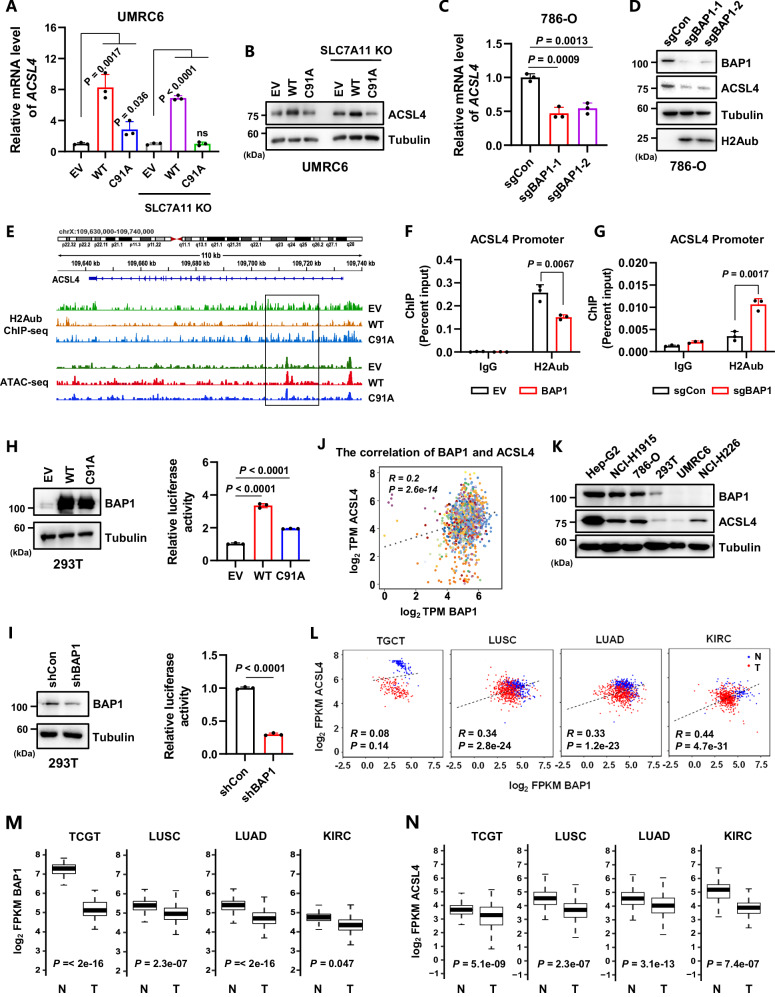
BAP1 upregulates ACSL4 expression and reduces H2Aub occupancy on the ACSL4 promoter. **A** ACSL4 expression levels in indicated UMRC6 cells were measured by RT-PCR. **B** Western blotting analysis of ACSL4 expression in SLC7A11-KO UMRC6 cells restored with empty vector, BAP1-WT, or C91A mutant cell lines. *n* = 4. mRNA and protein levels of indicated genes in indicated 786-O cells were measured by RT-PCR (**C**) and Western blotting (**D**). *n* = 4. **E** H2Aub ChIP-Seq occupancy profiles and accessible chromatin regions at the ACSL4 loci in indicated UMRC6 cells. **F** ChIP-qPCR confirming the lower H2Aub binding on the ACSL4 promoter in *BAP1* WT cells than in EV cells. **G** ChIP-qPCR showing the increased H2Aub binding on the ACSL4 promoter upon *BAP1* deficiency in 786-O cells. Western blotting and bar graphs showing ACSL4 promoter luciferase activity upon overexpression of BAP1-WT, C91A (**H**), or BAP1 knockdown (**I**) in HEK293T cells. *n* = 3. **J** The scatter plots show the positive correlation of BAP1 and ACSL4 expression in different cancer cell lines. **K** Immunoblotting analyses of BAP1 and ACSL4 protein levels in different cancer cell lines. *n* = 5. **L** The scatter plots show the positive correlation of BAP1 and ACSL4 expression in different cancer types. Box plots showing a comparison of BAP1 (**M**) or ACSL4 (**N**) expression levels in normal tissues (N) and corresponding tumor (T) samples. TGCT (testicular germ cell tumor), *n* = 154 independent tumor samples, *n* = 165 independent normal samples; LUSC (lung squamous cell carcinoma), *n* = 498 independent tumor samples, *n* = 338 independent normal samples; LUAD (lung adenocarcinoma), *n* = 515 independent tumor samples, *n* = 347 independent normal samples; KIRC (kidney renal clear cell carcinoma), *n* = 531 independent tumor samples, *n* = 100 independent normal samples. Error bars are mean ± SD. All *P* values were calculated using a two-tailed unpaired Student’s *t*-test. *n* ≥ 3 independent repeats unless specified. ns not significant (*P* > 0.05).

To identify the novel target downstream of BAP1 mediating its regulation of ferroptosis, we used RT-PCR to determine the mRNA levels of CP, ACSL4, and PRNP obtained from multi-omics analysis as mentioned above, while the level of SLC7A11 served as a positive control that was negatively regulated by BAP1. As shown in Figs. 3A and S3A, WT but not C91A could significantly upregulate the mRNA levels of ACSL4 and CP, consistent with our RNA-seq analysis. However, similar to SLC7A11, PRNP was also suppressed in the presence of WT rather than C91A (Fig. S3B, C), challenging the results obtained from transcriptomic analysis for PRNP expression (Fig. S1A). CP, known as ceruloplasmin to regulate iron homeostasis, has been demonstrated to suppress ferroptosis in hepatocellular carcinoma (HCC) cells [24], which prevents us from further studying the role of CP in promoting ferroptosis by BAP1. Given that ACSL4-mediated lipid biosynthesis is essential for ferroptosis induction, we therefore focused on exploring its potential regulation by BAP1. Then we checked the protein level and found that ACSL4 indeed was upregulated by WT but not its C91A mutant in UMRC6 cells regardless of SLC7A11 level (Fig. 3B). Conversely, reduction of BAP1 by CRISPR-Cas9 mediated gene knockout significantly repressed ACSL4 expression at mRNA and protein levels in BAP1-proficient renal cancer cell line 786-O (Fig. 3C, D). Similar results were also observed in MDA-MB-231 cells under overexpression or knockdown of BAP1 conditions (Fig. S3D, E). H2Aub ChIP-seq and ATAC-seq data revealed that restoration of BAP1-WT, but not BAP1-C91A, markedly decreased H2Aub occupancy and increased chromatin openness at both the promoter and gene body of ACSL4 (Fig. 3E). Next, we designed specific primers recognizing the ACSL4 gene promoter region and confirmed that BAP1 could negatively regulate the H2Aub level of ACSL4 gene by ChIP-qPCR in BAP1-restored UMRC6 cells or BAP1-knockout 786-O cells (Fig. 3F, G). In addition, overexpression of BAP1 dramatically increased and knockdown of BAP1 significantly decreased luciferase activities in the reporter plasmid with ACSL4 promoter region transfected into 293T cells, again proving the transcriptional regulation of ACSL4 by BAP1 (Fig. 3H, I). Previous studies showed that ACSL4 could be degraded by the ubiquitin-proteasome system [27]. To exclude that BAP1 upregulates ACSL4 through maintaining its protein stability, we treated cells with a protein-synthesis inhibitor, cycloheximide (CHX), and found that restoring BAP1 did not affect the stability of ACSL4. Meanwhile, MG132 treatment increased ACSL4 protein stability regardless of BAP1 status (Fig. S3F), suggesting that BAP1 is not involved in the post-translational regulation of ACSL4. It has been demonstrated that transcription factors, including HIF1α, c-Myc, and the STING/STAT1/IRF1 pathway, could regulate ACSL4 transcription (Fig. S3G) [28–30]. Our results indicated that although BAP1 potentially regulates these transcription factors at the transcriptional level (Fig. S3H–J), BAP1 could promote ACSL4 expression even in the HIF1α-, c-Myc, or STING-knockout cell lines (Fig. S3K–M), suggesting that HIF1α, c-Myc, or STING do not participate in the regulation of ACSL4 by BAP1. In addition, BAP1 and ACSL4 also exhibit a positive correlation in various types of cancer cells based on the Cancer Cell Line Encyclopedia (CCLE) database analysis (Figs. 3J and S3N). Consistent with this, we observed that cell lines with high expression of BAP1 tend to have relatively high ACSL4 levels (Fig. 3K). To explore the clinical relevance of the transcriptional regulation of ACSL4 by BAP1, we analyzed The Cancer Genome Atlas (TCGA) and GTEx datasets. As shown in Fig. 3L, it revealed a positive correlation between the expression levels of ACSL4 and BAP1 in testicular germ cell tumors (TGCT), lung squamous cell carcinoma (LUSC), lung adenocarcinoma (LUAD), and kidney renal clear cell carcinoma (KIRC). Moreover, both BAP1 and ACSL4 exhibited lower expression in tumors compared to the corresponding normal tissues (Fig. 3M, N), indicating a shared expression pattern in clinically relevant samples. In summary, the above data demonstrated that BAP1 positively regulates ACSL4 transcription in tumor cells.

### BAP1 promotes ferroptosis through upregulating ACSL4

**Fig. 4:**
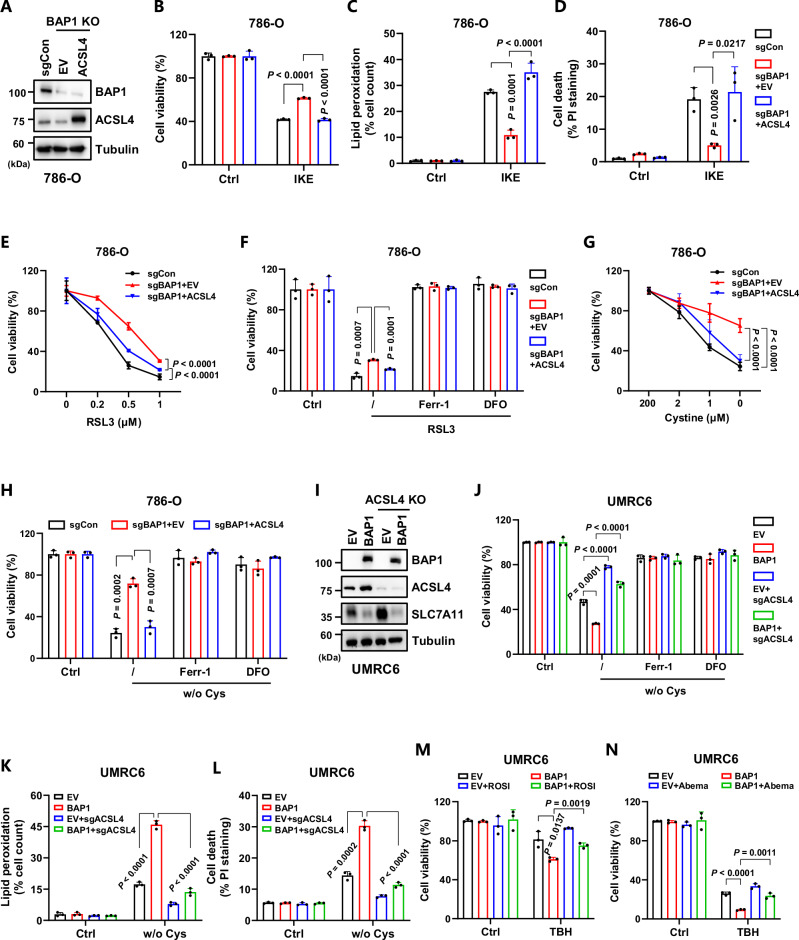
BAP1 regulates ferroptosis through ACSL4. **A** Protein levels of indicated genes in indicated 786-O cells were measured by Western blotting. *n* = 5. **B** Cell viability measured by CCK-8 assay in indicated cell lines treated with IKE (10 μM) for 24 h. **C** Lipid peroxidation was assessed by flow cytometry after C11-BODIPY staining in the indicated cells treated with IKE. **D** Cell death was measured in BAP1-KO 786-O cells restored with ACSL4 treated with IKE (10 μM) for 24 h. **E** Cell viability measured by CCK-8 assay in the indicated cell lines cultured in medium with different concentrations of RSL3. **F** Bar graph showing cell viability in indicated cells treated with RSL3 (1 μM) combined with 2 μM ferrostatin-1 (Ferr-1) or 100 μM deferoxamine (DFO). **G** Cell viability measured by CCK-8 assay in the indicated cell lines cultured in medium with different concentrations of cystine. **H** Bar graph showing cell viability in indicated cells treated with cystine limitation combined with 2 μM Ferr-1 or 100 μM DFO. **I** Western blotting analysis of ACSL4 expression in the corresponding cell lines. *n* = 4. **J** Bar graph showing cell viability in indicated cells treated with cystine limitation combined with 2 μM Ferr-1 or 100 μM DFO. **K** Bar graph showing lipid peroxidation in the indicated cells cultured in cystine-free medium. **L** Cell death was measured in the indicated UMRC6 cells cultured in cystine-free medium for 48 h. Bar graph showing cell viability in indicated cells treated with 10 μM TBH (ROS inducer) with or without rosiglitazone (10 μM) (**M**) or abemaciclib (1 μM) (**N**). Error bars are mean ± SD. All *P* values were calculated using a two-tailed unpaired Student’s *t*-test. *n* ≥ 3 independent repeats unless specified.

Even though SLC7A11 is involved in the regulation of ferroptosis by BAP1, the role of ACSL4 in BAP1-mediated ferroptosis remains elusive. We overexpressed ACSL4 in BAP1-KO 786-O cells, which exhibited a lower level of ACSL4 than that of control cells, and found that it rarely affected cell growth in vitro (Figs. 4A and S4A). Importantly, exogenous expression of ACSL4 resensitized BAP1-KO cells to IKE-induced cytotoxicity (Fig. 4B). Meanwhile, the lipid peroxidation level was dramatically restored in the same cell line upon IKE treatment (Figs. 4C and S4B). Moreover, cell death analysis was employed to confirm that ACSL4 overexpression indeed promoted IKE-induced ferroptotic cell death (Fig. 4D). Similarly, knockout of BAP1 promoted resistance to ferroptosis induced by Class II FINs like RSL3 and ML162, which could be reversed by overexpression of ACSL4 in BAP1-KO cells (Figs. 4E and S4C). However, these effects disappeared in the presence of ferroptosis inhibitors Ferr-1 or DFO (Figs. 4F and S4D). The effects of ACSL4 on the regulation of cell viability in BAP1-KO cells were also noted under the condition of cystine-low medium (Fig. 4G, H). Conversely, knockout of ACSL4 in UMRC6 cells significantly conferred resistance to ferroptosis induced by erastin, RSL3, or low-cystine medium, which was abrogated in the presence of ferroptosis inhibitors like Ferr-1 or DFO, but not Z-VAD-FMK and Nec-1s (Fig. S4E–J), again demonstrating the necessity of ACSL4 in promoting ferroptosis. Therefore, we restored BAP1 in ACSL4-KO cells which had a minimal influence on cell growth (Figs. 4I and S4K), and found that ACSL4 depletion alleviated the accumulation of lipid peroxidation and cell death caused by BAP1 under the condition of cystine deprivation, while such effects disappeared in the presence of ferroptosis inhibitors like Ferr-1 and DFO (Figs. 4J–L and S4L). In addition, we treated the cells with the ACSL4 inhibitors rosiglitazone (ROSI) or abemaciclib (Abema) in combination with ferroptosis inducer Tert-butyl hydroperoxide (TBH). Consistent with the effects of ACSL4 knockout, pharmacological inhibition of ACSL4 similarly restored cell viability that was suppressed by TBH (Fig. 4M, N). Besides, consistent with their effects on the regulation of ACSL4 by BAP1 (Fig. S3K–M), blocking HIF1α, c-Myc, or STING by their respective inhibitors PX-478, 10058-F4, or SN-011 rarely contributed to the regulation of BAP1-mediated ferroptosis (Figs. S3G and S4M–O). Collectively, these data demonstrated that BAP1 could promote ferroptosis through the upregulation of ACSL4.

### ACSL4-mediated lipid biosynthesis enhances ferroptosis sensitivity under the regulation of BAP1

**Fig. 5:**
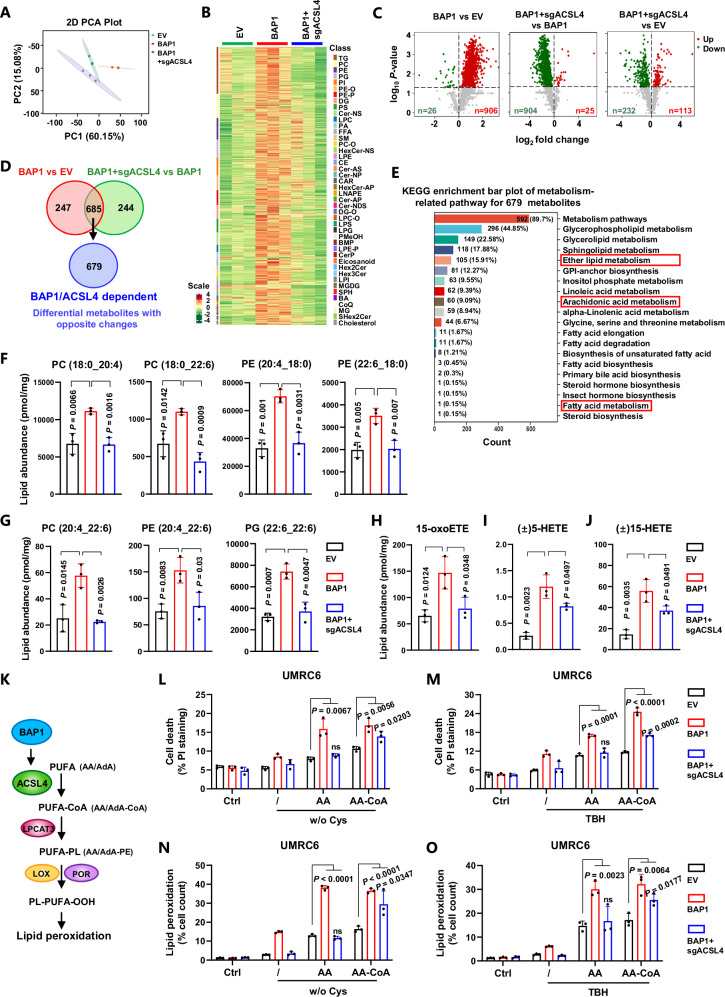
ACSL4-mediated lipid metabolism and ferroptosis sensitivity are regulated by BAP1. **A** Principal components analysis of indicated cell lines. **B** Heatmaps depicting the changes in the composition of each lipid subclass within the indicated cell lines. **C** Volcano plots representing lipidomic data from the indicated cell lines. The red and green dots represent lipid metabolites with at least 1-fold increase or decrease in indicated cells (*P* < 0.05). **D** Venn diagram illustrating the overlap of 679 BAP1/ACSL4-dependent metabolites between 932 and 929 differentially expressed metabolites (FC > 1, *P* < 0.05) in different comparison groups in UMRC6 cells. **E** KEGG enrichment bar plot of metabolism-related pathways for 679 metabolites. GPI Glycosylphosphatidylinositol. Bar graph showing lipid abundance of PL-PUFA_1s_ (**F**) and PL-PUFA_2s_ (**G**) in UMRC6-BAP1 and ACSL4-KO UMRC6 cells restored with BAP1-WT. Bar graph showing lipid abundance of 15-oxoETE (**H**), ( ± )5-HETE (**I**), and (±)15-HETE (**J**) in indicated cells. **K** Illustration of the ACSL4-mediated lipid metabolism regulation pathway. Cell death was measured in BAP1 OE UMRC6 cells lacking ACSL4 treated with cystine limitation (**L**) or TBH (10 μM) (**M**) combined with AA (1 μM) or AA-CoA (1 μM) for 6 h. Lipid peroxidation was assessed by flow cytometry after C11-BODIPY staining in indicated cells treated with cystine-free (**N**) or TBH (**O**) combined with AA or AA-CoA. Error bars are mean ± SD. All *P* values were calculated using a two-tailed unpaired Student’s *t*-test. *n* ≥ 3 independent repeats unless specified.

ACSL4 acts as a long-chain acyl-CoA synthetase and catalyzes the conjugation of long-chain PUFAs, including arachidonic acid (AA) and adrenic acid (AdA), with coenzyme A to produce AA-CoA or AdA-CoA, which are subjected to LPCAT3-mediated esterification to form phospholipids (PL), thereby increasing the incorporation of long-chain PUFAs into lipids needed for membrane structures within cells [16]. Due to the pivotal role of ACSL4 in lipid metabolism, we hypothesized that BAP1, as its upstream regulatory factor, may play a role in lipid metabolic reprogramming. We performed lipidomics analysis on the cell lines as described in Fig. 4I. The score plots of the principal component analysis from untargeted lipidomics were differentially clustered (Fig. 5A), suggesting distinct features of the analyzed cell lines. Importantly, the results revealed that BAP1 significantly upregulated multiple lipid subclasses (Fig. 5B). In particular, the levels of triacylglycerols (TG), phosphatidylcholines (PC), and phosphatidylethanolamines (PE) were all markedly increased (Fig. S5A–C and Supplementary Material). Importantly, these upregulated lipids by BAP1 were significantly decreased in the absence of ACSL4 (Fig. 5B, C), suggesting that BAP1 promotes this lipid biosynthesis at least partially through upregulating ACSL4. Notably, we found that among the 932 differentially metabolized lipids regulated by BAP1, 679 lipid metabolites were counter-regulated upon ACSL4 knockout (Fig. 5D). Specifically, ACSL4 knockout suppressed the 671 lipid metabolites that were upregulated by BAP1, suggesting that BAP1 promotes lipid metabolism mainly through ACSL4. Next, BAP1/ACSL4-dependent regulated lipids were grouped according to their KEGG classification, and metabolites involved in the ether lipid metabolism, polyunsaturated FA metabolism as well as AA metabolism were noted (Fig. 5E). Specifically, different long-chain PUFA-containing phospholipid species which have been reported to be the most vulnerable to peroxidation include PL-PUFA_1s_ and PL-PUFA_2s_ [31], were significantly upregulated by BAP1, but this effect was largely abolished after silencing ACSL4 (Figs. 5F, G and S5D). Moreover, the lipidomic results indicated that knockout of ACSL4 counteracted the upregulation of oxidized derivatives of AA like 15-oxoETE, (±)5-HETE, and (±)15-HETE in BAP1 re-restored cells (Fig. 5H–J). Given the key role of ACSL4-mediated PUFA biosynthesis in the regulation of ferroptosis (Fig. 5K), we treated cells with cystine deprivation or TBH in combination with the substrate AA or catalytic product AA-CoA of ACSL4. Consistent with previous conclusions, BAP1 sensitized cells to ferroptosis induced by TBH or cystine limitation, while knockout of ACSL4 effectively relieved the cell death (Figs. 5L, M and S5E). Upon treatment with the ACSL4 substrate AA, the resulting accumulation of polyunsaturated FAs supplied abundant precursors for lipid peroxidation, which in turn exacerbated the ferroptosis-sensitizing effect of BAP1. However, this combined treatment failed to facilitate accumulation of lipid peroxidation and cell death in ACSL4-deficient cells (Fig. 5L–O). Co-treatment with AA-CoA, the product of ACSL4, partly counteracted the impact of ACSL4 inhibition on lipid generation, consequently reversing the effect of ACSL4 knockout and promoting BAP1-mediated ferroptosis in those cells (Fig. 5L–O). Taken together, our data suggested that BAP1 promotes ferroptosis at least partly through BAP1-mediated upregulation of ACSL4 expression as well as ACSL4-mediated lipid metabolism.

### ASXL1 is required for the regulation of gene expression and ferroptosis by BAP1

**Fig. 6:**
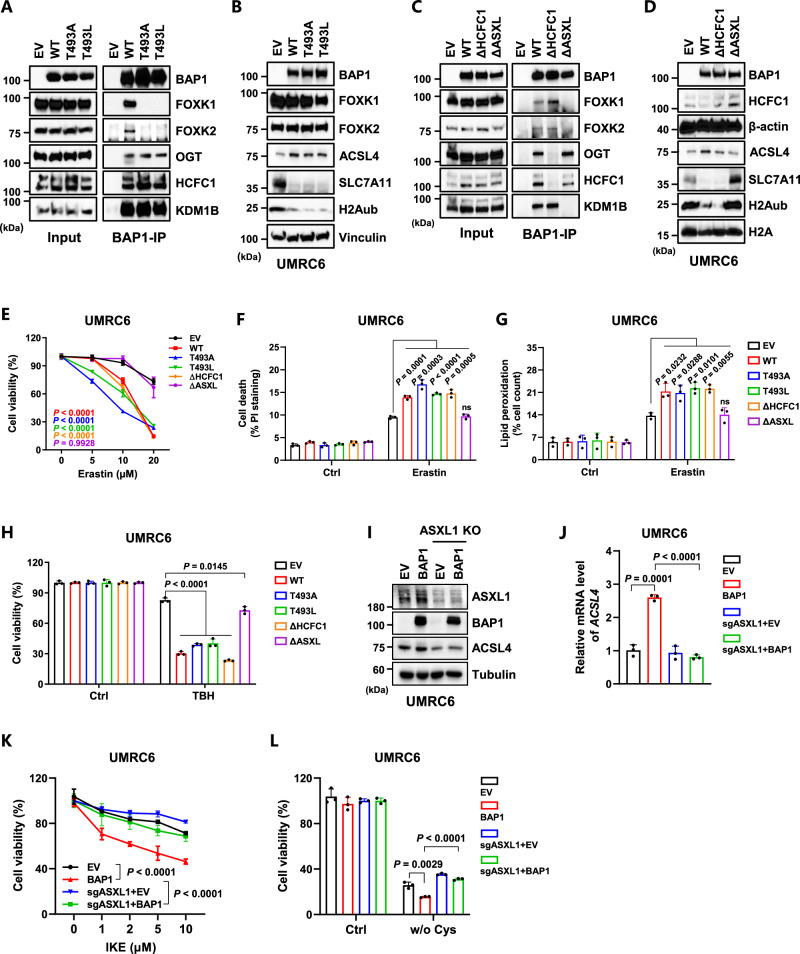
ASXLs are required for the regulation of gene expression and ferroptosis by BAP1. **A**–**D** Interactions between indicated proteins and BAP1 were verified by Western blotting in indicated cells (**A**, **C**). Western blotting analysis of indicated proteins in BAP1-WT and mutations UMRC6 cell lines (**B**, **D**). **E** Cell viability in indicated cells was measured after treatment with different concentrations of erastin for 30 h. **F** Bar graph showing cell death of the indicated cells upon 10 μM erastin treatment for 24 h. **G** Lipid peroxidation in the indicated cells after treatment with 10 μM erastin for 24 h was assessed by flow cytometry after C11-BODIPY staining. **H** Cell viability of indicated cells was measured after treatment with TBH (10 μM) for 6 h. Western blotting (**I**) and RT-PCR (**J**) analysis of ACSL4 expression in ASXL1-KO UMRC6 cells restored with BAP1. **K** Cell viability in indicated cells was measured after treatment with different concentrations of IKE for 24 h. **L** Bar graph showing cell viability in indicated cells treated with cystine limitation. Error bars are mean ± SD. All *P* values were calculated using a two-tailed unpaired Student’s *t*-test. *n* ≥ 3 independent repeats unless specified. ns not significant (*P* > 0.05).

Serving as the core component in the PR-DUB complex, BAP1 regulates the expression of target genes not only through its deubiquitinase activity but also with the assistance of other associated molecules, including FOXK1/2, KDM1B, OGT, HCFC1, and ASXL1/2 [3, 4, 21, 32]. To further explore the role of these interacting proteins in the regulation of gene expression and ferroptosis by BAP1, we constructed corresponding BAP1 mutants that lose the protein-interacting abilities based on the reported sites or regions of BAP1 that mediate its interaction with other partners [33, 34]. The T493A and T493L mutants of BAP1 failed to interact with FOXK1 or FOXK2 as verified by pull-down of BAP1 protein followed by Western blotting (Fig. 6A). Deletion of the amino acids from 363 to 366 within BAP1 (ΔHCFC1) lost the ability to interact with HCFC1, while removal of the amino acids from 666 to 669 within BAP1 (ΔASXL) lost the ability to interact with ASXL family proteins like ASXL1 and ASXL2, even though ΔASXL mutant also couldn’t interact with FOXK1/2 and KDM1B either (Figs. 6C and S6A, B). As a result, while other mutants retained function, ΔASXL lost the ability to upregulate ACSL4 expression and suppress SLC7A11 and H2Aub (Fig. 6B–D), suggesting that BAP1 regulates gene expression and decreases H2Aub through its interaction with ASXL family proteins. Next, we observed that restoring the expression of T493A, T493L, and ΔHCFC1, but not the ΔASXL mutant of BAP1, sensitized BAP1-deficient UMRC6 cells to erastin-induced ferroptosis to a comparable level with WT (Figs. 6E and S6C). Consistently, the cell death and lipid peroxidation levels were also significantly elevated in cell lines restored with these BAP1 mutants except ΔASXL (Figs. 6F, G and S6D). Besides, we treated the cells with another ferroptosis inducer, TBH, and further demonstrated that the ΔASXL mutant lost its function to promote ferroptosis compared to WT and other mutants of the BAP1 protein (Fig. 6H). Notably, we didn’t observe a huge difference in terms of the effects of these mutants on cell growth (Fig. S6E, F). To further determine the role of ASXL1 and ASXL2 in BAP1 regulation of gene expression and ferroptosis (ASXL3 is excluded due to its tissue-specific expression pattern in vivo), we generated ASXL1-KO and ASXL2-KO cells and found that knockout of ASXL1 or ASXL2 differentially changed ACSL4 expression and ferroptosis sensitivity in UMRC6 cells without BAP1 expression (Fig. S6G–K). Importantly, restoring BAP1 expression failed to upregulate ACSL4 and to promote ferroptosis in ASXL1-KO cells (Fig. 6I–L), suggesting that ASXL1 is required for the regulation of ACSL4 expression and ferroptosis by BAP1. Besides, BAP1 couldn’t further increase ACSL4 expression in ASXL2-KO cells, possibly due to the fact that ASXL2 deletion already dramatically increased ACSL4 expression (Fig. S6H, L–N). In summary, our data suggest that ASXL1 is indispensable for the regulation of gene expression and ferroptosis by BAP1.

## Discussion

**Fig. 7:**
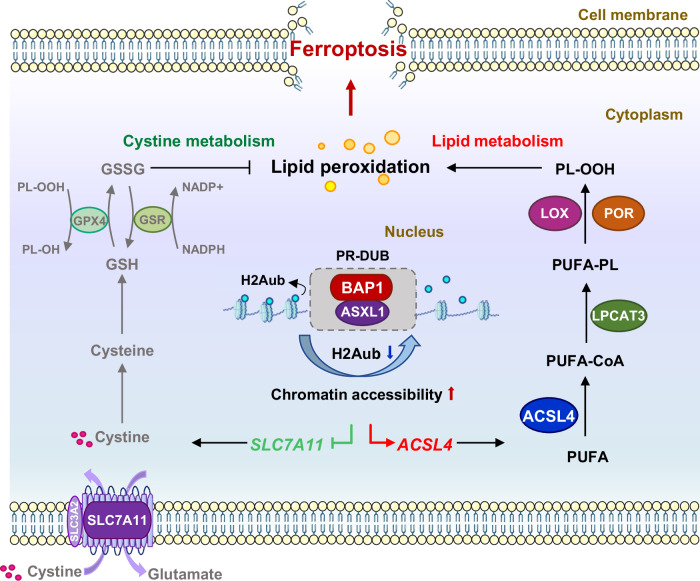
Proposed working model.

Our research demonstrated that BAP1 promotes the expression of ACSL4 through epigenetic regulation of H2Aub at the genomic level, further linking ACSL4-mediated lipid metabolism to BAP1-mediated ferroptosis (Fig. 7). Notably, our integrated multi-omics analysis proposed a paradigm where the BAP1-dependent deubiquylation of H2Aub enhances the degree of chromatin spatial openness. We showed that wild-type rather than catalytic-dead mutant BAP1 drives extensive changes in chromatin accessibility, which is accompanied by dysregulation of gene expression at the transcriptional level (Fig. 1G, H). Subsequently, we identified a novel transcriptional target, ACSL4, among these dysregulated genes, which promotes lipid biosynthesis and ferroptosis. Notably, knockout of ACSL4 abrogated ferroptosis sensitivity caused by BAP1 even in the SLC7A11-deficient cells (Fig. 4), suggesting ACSL4-mediated lipid metabolism as a parallel mechanism underlying the regulation of ferroptosis by tumor suppressor BAP1. Moreover, we examined the role of core components within the PR-DUB complex in the regulation of its transcriptional targets ACSL4 and SLC7A11, and demonstrated that ASXL family proteins are required for BAP1 regulation of H2Aub deubiquitination, gene expression, and ferroptotic cell death. These findings offer additional insights for further exploring the mechanisms by which tumor suppressor BAP1 regulates gene transcription as well as metabolism at the epigenetic level during tumor development.

Our previous study has confirmed that BAP1 promotes ferroptosis and suppresses tumor growth through inhibiting SLC7A11 transcription and subsequent cystine uptake in a H2Aub-dependent manner, which links BAP1 to SLC7A11-mediated cystine metabolism and ferroptosis regulation [14]. The cystine/glutamate antiporter SLC7A11 responds to various stress conditions like oxidative stress and nutrient stress, thus adequately adjusting its expression level through key transcription factors like NRF2 and ATF4 to adapt to the challenging environments [35]. The findings of BAP1 regulation of SLC7A11-mediated cystine metabolism indicate the important role of tumor suppressor BAP1 in controlling tumor development with dynamic environmental changes. In the current study, we identified ACSL4 as a key transcriptional target epigenetically regulated by BAP1 and demonstrated that ACSL4-mediated lipid biosynthesis contributes to ferroptosis driven by BAP1, which expands our understanding of the metabolic regulation of tumor cells by BAP1. Given the heterogeneity of tumors, certain types of tumors rely more heavily on lipid metabolism to supply both building blocks for biomembrane synthesis and energy production, ultimately supporting their rapid proliferation [36, 37]. ACSL4-mediated lipid metabolism could be considered an additional targeted susceptibility for BAP1-associated cancers in which lipid biosynthesis constitutes a major additive metabolic vulnerability. Thus, these findings suggest that BAP1 promotes ferroptosis through both SLC7A11-dependent cystine metabolism and ACSL4-dependent lipid metabolism in tumor cells.

Chromatin accessibility changes dynamically in response to both external stimuli and internal epigenetic regulators, and emerging evidence suggests that histone modifications like phosphorylation, methylation, acetylation, lactylation, and ubiquitination are involved in this process [38]. Monoubiquitination of H2A (H2AK119ub1) or H2B (H2BK123ub1) seems most prevalent, although other modification sites prone to be monoubiquitinated remain largely elusive [39]. H2Aub is conceived as a switch to turn off gene transcription, thus leading to gene silencing [40]. However, little is known about whether monoubiquitination of H2A represses gene expression through changing chromatin accessibility. Multi-omics analyses from different Arabidopsis PcG mutants indicate that monoubiquitylation of H2A is associated with a less accessible chromatin, despite the fact that increased chromatin accessibility caused by the loss of PcG activities is not necessarily accompanied by transcriptional activation [41]. In addition, loss of BAP1 via CRISPR/Cas9 in normal human cholangiocyte organoids reduces the expression of junctional and cytoskeleton components and unexpectedly leads to increased chromatin accessibility, suggesting that BAP1 regulates gene expression in a context-dependent manner [42]. To further examine the role of BAP1 in the regulation of chromatin accessibility and gene expression, we employed BAP1-deficient renal carcinoma cell line UMRC6 and introduced exogenous BAP1 followed by multi-omics analyses, demonstrating that restoration of BAP1 dramatically increased the openness at the genomic level, though it caused similar effects on the up- or down-regulation of gene transcription (Fig. 1G, H), indicating that BAP1 promotes chromatin accessibility at least partly through H2Aub deubiquitylation. However, the realistic impact on target gene expression appears to be uncertain, and whether specific transcription factors are involved remains to be further investigated.

Lipids not only serve as the essential components of cellular biomembranes but also participate in the remodeling of cellular functions, which are crucial for maintaining cellular homeostasis, regulating tumor progression, and the tumor microenvironment [43, 44]. However, various reports suggest an inconsistent role of ACSL4-mediated lipid metabolism in tumor progression. Hepatocyte-specific deletion of ACSL4, which prevents ferroptotic cell death in hepatocytes, does not increase the formation of HCC. Instead, ACSL4-dependent ferroptosis has an unanticipated cancer-promoting effect during HCC formation, possibly due to aggravated liver damage [45]. Similarly, ACSL4 emerged as a critical biomarker of hepatoblastoma (HB) and drove HB cell proliferation both in vitro and in vivo [46]. On the other hand, ACSL4-mediated lipid metabolism can also induce immunogenic tumor ferroptosis and enhance CD8⁺ T cell (CTL)-mediated antitumor immunity, which represents a potential anti-cancer approach [25]. Intriguingly, independent findings demonstrate that ACSL4 enhances membrane fluidity, promotes cell invasiveness, and facilitates metastatic extravasation of ovarian and breast cancer cells [47–49]. Our findings demonstrate that BAP1 promotes ferroptosis through ACSL4-mediated lipid biosynthesis, indicating that ACSL4 might contribute to natural tumor suppression mediated by the tumor suppressor. However, whether the status of ACSL4 plays a vital role in tumorigenesis under the condition of BAP1 deficiency or inactivation in vivo remains to be further explored. Besides, BAP1, though regarded as a tumor suppressor, is reported to promote tumor cell invasion and metastasis in breast cancers [50], which prompts further investigation on the effect of ACSL4 in BAP1 regulation of tumor metastasis in the future.

In summary, our study uncovered a novel pathway by which BAP1 regulates ferroptosis besides cystine metabolism. Moreover, the activation of BAP1 regulates various lipid components, including not only the “fuel” for ferroptosis but also precursors of lipid droplets (LDs), which store FAs as triacylglycerols. LDs alleviate oxidative stress by controlling PUFA transportation or promote ferroptosis through lipophagy and selective degradation of PUFA-enriched LDs [51, 52]. A key emerging question is whether dynamic changes in LDs contribute to BAP1-mediated lipid metabolic regulation, which will be a focus of our subsequent research. Therefore, the above observation inspires further research to explore other pathways through which BAP1 regulates tumor cell metabolism and its role in tumor suppression. Our recent findings demonstrate that BAP1 promotes glucose dependency and inhibits glucose limitation-induced disulfidptosis in tumor cells [53], adding complexity to the metabolic regulation of tumor progression by BAP1. Thus, the exact contribution of BAP1 and its transcriptional targets, like ACSL4 or SLC7A11, to tumor progression should be examined in particular metabolic contexts or tumor types (genetic background), which should reveal distinct outcomes in terms of regulation of cell death and tumor progression. It will be intriguing to test this hypothesis in future research, providing new theoretical foundations and ideas for clinical treatment of BAP1-TPDS.

## Materials and methods

### Cell culture studies

HEK293T (CRL-3216), 786-O (CRL-1932) cell lines were obtained from ATCC. The UMRC6 cell line was originally purchased from Sigma (#08090513, 2017). NCI-H226 cell was kindly provided by the Stem Cell Bank, Chinese Academy of Sciences. MDA-MB-231 was provided by Dr. Lianying Jiao at Xi’an Jiaotong University. All cell lines were maintained and cryopreserved in our laboratory and were routinely tested for mycoplasma contamination. The cell lines were cultured in DMEM or RPMI-1640 supplemented with 10% FBS and 1% penicillin-streptomycin. For cystine deprivation experiments, cells were cultured in cystine-free DMEM supplemented with 10% dialyzed FBS.

### Constructs and stable cell line generation

To generate stable overexpression or knockout cell lines, HEK293T cells were transfected with the corresponding recombinant overexpression or LentiCRISPR V2-sgRNA constructs, along with the third-generation lentiviral packaging plasmids psPAX2 and pMD2.G, using polyethylenimine linear (MW 40000) reagent (Yeasen, 40816ES02, China) according to the manufacturer’s instructions. The lentivirus particles in the medium were collected and filtered 48 h after transfection. Target cell lines were then infected in the presence of polybrene transfection reagent (Solarbio, H8761, China). Stable cell lines were selected with puromycin (Solarbio, P8230, China) after virus infection. Gene knockout was performed by sgRNAs and CRISPR/Cas9 technology. CRISPR-mediated knockout plasmids containing guide RNAs were generated in LentiCRISPR V2 (Addgene, #52961, USA) according to the protocol. Flag-BAP1 and mutant BAP1, which were generated by PCR mutagenesis using the QuikChange II XL Site-Directed Mutagenesis Kit (Agilent, 200523, USA) for amino acid substitutions according to the manufacturer’s instructions, were cloned into the pLVX-puro plasmid. All constructs were confirmed by DNA sequencing. To generate shRNA-knockdown cell lines, lentiviral transduction with shRNA vectors was conducted as described above. After 72 h of antibiotic selection, expression levels of target genes were determined by immunoblotting with corresponding antibodies. The sequences of the primers used in PCR mutagenesis, sgRNAs, and shRNAs used in this study are listed in Supplementary Material.

### Reagents and antibodies

The reagents were purchased as follows: Z-VAD-FMK (T7020), PX-478 (T6961), 10058-F4 (T3048), Abemaciclib (T2381), Deferoxamine mesylate (T1637) were bought from TargetMol (Shanghai, China); TBH (B106035) was bought from Macklin (Shanghai, China); Arachidonoylcoenzyme A lithium salt (A293430) was purchased from Aladdin (Shanghai, China); BODIPY 581/591 C11 (D3861) was bought from Thermo Fisher Scientific (Waltham, Massachusetts, USA); L-cystine (A610088), propidium iodide (A601112) were obtained from Sangon Biotech (Shanghai, China); Necrostain 2 racemate (S8641), RSL3 (S8155), ML162 (S4452), erastin (S7242), Imidazole ketone erastin (S8877) and Liproxstain-1 (S7699) were purchased from Selleck (Houston, Texas, USA); SN-011 (HY-145010), Rosiglitazone (HY-17386), AA (HY-109590) were purchased from MedChemExpress (Monmouth Junction, New Jersey, USA). All reagents were dissolved according to the manufacturer’s instructions.

The primary antibodies used for Western blotting assays were as follows: β-Tubulin (66240-1-Ig), Vinculin (66305-1-Ig), HIF1α (20960-1-AP), STING (19851-1-AP), GAPDH (HRP-60004) were bought from Proteintech (Wuhan, China); ACSL4 (sc-271800) was purchased from Santa Cruz Biotechnology (Dallas, Texas, USA); β-actin (AC004) was obtained from ABclonal Technology (Wuhan, China); BAP1 (#13271S), SLC7A11 (#12691), H2Aub (#8240S), H2A (#12349S), ASXL1 (#52519s), ASXL2 (#71257s), OGT (#5368), Flag-tag (#14793), Myc-tag (#2276) were obtained from Cell Signaling Technology (Danvers, Massachusetts, USA); c-Myc (#0912-2) was obtained from HUABIO (Hangzhou, China); FOXK1 (ab18196) and KDM1B (ab193080) were obtained from Abcam (Cambridge Biomedical Campus, Cambridge, UK); FOXK2 (A301-730A) and HCFC1 (A301-399A) were bought from Bethyl Laboratories (Montgomery, Texas, USA).

### Cell viability/death/proliferation assays

For the cell viability assay, 5000 cells per well were seeded in a 96-well plate 1 day before treatment. After treating the cells with appropriate drugs, the medium in each well was replaced with fresh medium containing Cell Counting Kit-8 reagent (EnoGene, E1CK-000208-1, China). After 1 h of incubation at 37 °C, the absorbance of each well was measured with a microplate reader (Thermo Scientific, Waltham, Massachusetts, USA) at a wavelength of 450 nm.

Cell death was measured by PI staining using a flow cytometer (BD Biosciences, Franklin Lakes, New Jersey, USA). Cells were seeded in a 12-well plate 1 day before treatment. After treating the cells with the designed drugs, cells were trypsinized and collected in tubes, washed with PBS, and stained with 2 μg/mL PI (Sangon, Biotech, A610088) in PBS. Dead cells (PI-positive cells) were analyzed and plotted by FlowJo 10 software.

For the cell proliferation assay, 2000 cells per well were seeded in a 96-well plate. Viability was measured 24 h later (designated as day 0) using the CCK-8 reagent and subsequently every 24 h. The cell proliferation curve was presented as the fold change from day 0 to day 4.

### Lipid peroxidation assay

Lipid peroxidation levels were measured as previously described [54]. Briefly, cells were seeded in a 6-well plate 1 day before treatment. After treating the cells with the designed drugs, the cells were incubated with fresh medium containing 5 μM BODIPY 581/591 C11 dye. After incubation for 30 min at 37 °C, cells were washed with PBS and trypsinized, then collected in tubes. Cells were subjected to flow cytometry analysis to detect the fluorescence intensity of BODIPY 581/591 C11 using a flow cytometer (BD Biosciences, Franklin Lakes, New Jersey, USA).

### GSH assay

The concentrations of glutathione (GSH) and oxidized glutathione (GSSG) were measured using a GSH and GSSG Assay Kit (Beyotime, S0053, China). The assay principle is based on the reaction of GSH with DTNB (5,5′-dithiobis-2-nitrobenzoic acid) to generate TNB (2-nitro-5-thiobenzoic acid), which exhibits a yellow color detectable by absorbance. Total glutathione content is determined by reducing GSSG to GSH in the presence of glutathione reductase, thereby enabling the measurement of TNB formation from all available glutathione. To specifically quantify GSSG, GSH in the sample was first removed. The remaining GSSG was then reduced to GSH and measured as described. The GSH content is calculated by subtracting the GSSG content from the total glutathione. All procedures were performed in accordance with the manufacturer’s instructions.

### Real-time PCR and ChIP-qPCR

For real-time PCR, total RNA of each sample was extracted using Trizol reagent (ABclonal, RK30129). Then, 2 μg of RNA was subjected to reverse transcription into cDNA using ABScript III RT Master Mix for qPCR (ABclonal, RK21428). Real-time PCR was performed in a 20 μL reaction mixture system by using 2× Universal SYBR Green Fast qPCR Mix (ABclonal, RK21203). β-actin or GAPDH was used as an internal control. ChIP experiments were performed using a SimpleChIP Plus Enzymatic Chromatin IP Kit (Cell Signaling Technology, 9005) according to the manufacturer’s instructions. Briefly, after crosslinking of proteins to DNA, the chromatin was digested and sonicated. Antibodies against H2Aub and control IgG were used for immunoprecipitation. The enriched DNA fragments captured by antibodies were examined using real-time PCR. The signal relative to input was evaluated using a formula from the manufacturer’s protocol as follows: percent input = 2% × 2^(*C*[*T*]2% input sample−*C*[*T*]IP sample)^, where *C*[*T*] = average threshold cycle of PCR reaction. The sequences of primers used for real-time PCR and ChIP-qPCR are listed in Supplementary Material.

### Luciferase reporter assay

The ACSL4 promoter was amplified from genomic DNA extracted from UMRC6 cells using AFTSpin Blood/Tissue/Cell Fast DNA Extraction Kit (ABclonal, RK30110) and cloned into pGL4.35 luciferase reporter vectors. Primers used to clone the promoter region of ACSL4 are listed in Supplementary Material. The luciferase reporter assay was conducted using a Dual Luciferase Reporter Gene Assay Kit (Beyotime, RG027) according to the manufacturer’s instructions. Briefly, HEK293T cells were transfected with the appropriate plasmids (including pGL4.35-ACSL4 promoter reporter plasmid and pRL-TK internal control plasmid) for 48 h, washed with PBS, and lysed at room temperature. Cell lysates were transferred to a 96-well plate for subsequent luciferase activity measurement. Luminescence was measured using a Gen5 software version 3.08 microplate reader (BioTek).

### Immunoprecipitation and Western blotting

For co-immunoprecipitation to detect protein-protein interactions in vitro, cell lysates were incubated with Anti-Flag/Myc Magnetic Beads (Beyotime, P2115; P2118) as indicated, overnight at 4 °C. After washing the beads three times with lysis buffer, the bound immune complexes were eluted and analyzed by SDS-PAGE.

For Western blotting, cells were lysed in NP-40 buffer (150 mM sodium chloride, 1% NP-40, 50 mM Tris, pH 8.0) containing protease and phosphatase inhibitors. The lysates were sonicated, centrifuged, and the supernatant was collected. After determining the protein concentration and denaturing the samples (95 °C, 5 min), equal amounts of protein were separated by SDS-PAGE and transferred to a PVDF membrane. The membrane was blocked with 5% skim milk and then incubated sequentially with primary and secondary antibodies. Signals were detected using an enhanced chemiluminescence system (Baygene Biotech, Shanghai, China). Full and uncropped Western blots are provided as Supplementary Material.

### ATAC-seq and analysis

The principle of this experiment is as follows: the method uses a hyperactive Tn5 transposase to simultaneously fragment and tag open chromatin regions by inserting sequencing adapters. Subsequent PCR amplification results in the creation of a sequencing library, which allows for the comprehensive identification of open chromatin regions under specific space-time conditions. For UMRC6-EV, -WT, -C91A cells, approximately 1 × 10^6^ living cells per cell line were taken for library construction. The cells were lysed in ATAC-seq lysis buffer to obtain the nuclei, and the transposase-treated libraries were constructed using the DNA library kit for Illumina. PCR was performed to amplify the libraries. The libraries were sequenced on Illumina NovaSeq to generate paired-end reads (2 × 42 bp in length). ATAC-seq was performed in three biological replicates.

The raw data were filtered by cutadapt (version 1.18) [55] to remove adapter-polluted or low-quality reads with the following parameters “-a CTGTCTCTTATACACATCT -g AGATGTGTATAAGAGACAG -A CTGTCTCTTATACACATCT -G AGATGTGTATAAGAGACAG -m 35 --max-n 0.1 -q 10”. For each sample, all reads were aligned to the human reference genome (hg38) using Bowtie2 (version 2.5.1) [56] with the parameters “-X 2000 --local --mm --threads 32.” The bam files were generated with samtools (version 1.16.1) [57]. Peaks were called using MACS2 (version 2.2.6) [58] with the parameters “-p 1e-3 --keep-dup all --broad.” The peaks were annotated by the R package ChIPseeker (version 1.32.1) [59]. Chromatin accessibility changes following BAP1 restoration in UMRC6 cells were analyzed using ChIPseeker. Differential accessibility was defined by the thresholds of a *P* < 0.1 and a minimum 1.5-fold change (genes with *P* < 0.1 were considered as candidates for further validation). Statistical significance was assessed using Student’s *t*-test.

### RNA-seq and analysis

RNA sequencing was performed in our previous study [14]. The differentially expressed genes upon restoring BAP1 in UMRC6 cells were defined by a cutoff of FDR  <  0.05 with at least a 1.5-fold change.

### ChIP-seq and analysis

ChIP-seq was performed in our previous study [14]. The genes with differential H2Aub occupancy upon restoring BAP1 in UMRC6 cells were defined by a cutoff of at least a 1.6-fold change.

### Lipidomics analysis

Briefly, UMRC6 cells were cultured as described previously. The cells were then washed three times with ice-cold PBS, harvested by scraping into a centrifuge tube, and stored on dry ice. The resulting cell pellet was homogenously resuspended in 100 μL of ultrapure water-based extraction buffer supplemented with protease inhibitors, PMSF, and EDTA. Subsequently, 50 μL of the cell suspension was transferred to 500 μL of lipid extraction solution containing an internal standard mixture. After vortexing for 2 min and sonicating for 5 min, 100 μL of water was added to the mixture. The sample was vortexed again for 1 min and centrifuged at 12,000 rpm and 4 °C for 10 min. Following centrifugation, 200 μL of the supernatant was carefully transferred to a labeled centrifuge tube and concentrated. The concentrate was reconstituted in 200 μL of lipid reconstitution solution for LC-MS/MS analysis. Meanwhile, the remaining 50 μL of cell suspension underwent three cycles of snap-freezing in liquid nitrogen and thawing, followed by centrifugation at 12,000 rpm for 10 min. Then, lipid contents were detected by MetWare (http://www.metware.cn/) based on the AB Sciex QTRAP 6500 LC-MS/MS platform. The analyses were performed using the Metware Cloud, a free online platform for data analysis.

### Bioinformatic analysis

The gene expression data of BAP1 and ACSL4 in 4 cancer types (TGCT, LUSC, LUAD, and KIRC) were obtained from TCGA, while the expression data for normal samples were from the GTEx database. The gene expression of BAP1 and ACSL4 in different cell lines was obtained from the CCLE. All the above data were generated using the UCSC Xena Browser (http://xena.ucsc.edu/).

Statistical analyses were carried out in the R package for Figs. 1B, E, I, J and 3M, N. Differential comparisons were evaluated by a two-tailed Student’s *t*-test. Boxplots display the median (central line), the interquartile range (IQR; box limits at 25th and 75th percentiles), and whiskers extending to 1.5 × IQR. For GO biological process and KEGG pathway enrichment (Figs. 2A, S1B, C and S2A), *P* values were computed by Fisher’s exact test and subsequently adjusted for multiple testing by the Benjamini–Hochberg procedure. Pearson’s correlation (two-sided) was used to assess gene-gene relationships in Figs. 3J, L and S3N.

### Statistical analysis

All results presented in this study were consistently observed in at least three independent experiments. Western blot analyses were independently repeated a minimum of three times, with comparable outcomes across replicates. Data are presented as bar graphs displaying mean ± SD. Statistical analyses and graph generation were performed using GraphPad Prism software. Differences were considered statistically significant at *P* < 0.05; ns denotes not significant (*P* > 0.05). Comparisons between two groups were analyzed using a two-tailed unpaired Student’s *t*-test, while comparisons among three or more groups were conducted using two-way ANOVA.

## Supplementary information

Supplementary information

Supplementary Material-Table 1

Supplementary Material-Table 2

Supplementary Material-Table 3

Supplementary Material-uncropped western blots

## Data Availability

ATAC-seq data supporting the findings of this study have been deposited in the Genome Sequence Archive [60] in the National Genomics Data Center [61], China National Center for Bioinformation/Beijing Institute of Genomics, Chinese Academy of Sciences (GSA-Human: HRA013291), which are publicly accessible at https://ngdc.cncb.ac.cn/gsa-human. Other data supporting the findings of this study are available from the corresponding author upon reasonable request.

## References

[1] Barbour H, Daou S, Hendzel M, Affar EB. Polycomb group-mediated histone H2A monoubiquitination in epigenome regulation and nuclear processes. Nat Commun. 2020;11:5947. 33230107 10.1038/s41467-020-19722-9 PMC7683540

[2] Carbone M, Harbour JW, Brugarolas J, Bononi A, Pagano I, Dey A, et al. Biological mechanisms and clinical significance of BAP1 mutations in human cancer. Cancer Discov. 2020;10:1103–20. 32690542 10.1158/2159-8290.CD-19-1220 PMC8006752

[3] Daou S, Hammond-Martel I, Mashtalir N, Barbour H, Gagnon J, Iannantuono NVG, et al. The BAP1/ASXL2 histone H2A deubiquitinase complex regulates cell proliferation and is disrupted in cancer. J Biol Chem. 2015;290:28643–63. 26416890 10.1074/jbc.M115.661553 PMC4661380

[4] Yu H, Mashtalir N, Daou S, Hammond-Martel I, Ross J, Sui G, et al. The ubiquitin carboxyl hydrolase BAP1 forms a ternary complex with YY1 and HCF-1 and is a critical regulator of gene expression. Mol Cell Biol. 2010;30:5071–85. 20805357 10.1128/MCB.00396-10 PMC2953049

[5] Lalloo F, Kulkarni A, Chau C, Nielsen M, Sheaff M, Steele J, et al. Clinical practice guidelines for the diagnosis and surveillance of BAP1 tumour predisposition syndrome. Eur J Hum Genet. 2023;31:1261–9. 37607989 10.1038/s41431-023-01448-z PMC10620132

[6] Faubert B, Solmonson A, DeBerardinis RJ. Metabolic reprogramming and cancer progression. Science. 2020;368:eaaw5473. 10.1126/science.aaw5473 PMC7227780 32273439

[7] Pavlova NN, Thompson CB. The emerging hallmarks of cancer metabolism. Cell Metab. 2016;23:27–47. 26771115 10.1016/j.cmet.2015.12.006 PMC4715268

[8] Zhang J, Chen M, Yang Y, Liu Z, Guo W, Xiang P, et al. Amino acid metabolic reprogramming in the tumor microenvironment and its implication for cancer therapy. J Cell Physiol. 2024;239:e31349. 38946173 10.1002/jcp.31349

[9] Yang K, Wang X, Song C, He Z, Wang R, Xu Y, et al. The role of lipid metabolic reprogramming in tumor microenvironment. Theranostics. 2023;13:1774–808. 37064872 10.7150/thno.82920 PMC10091885

[10] Guo D, Ji X, Xie H, Ma J, Xu C, Zhou Y, et al. Targeted reprogramming of vitamin B3 metabolism as a nanotherapeutic strategy towards chemoresistant cancers. Adv Mater. 2023;35:e2301257. 37262365 10.1002/adma.202301257

[11] Jiang B, Zhang J, Zhao G, Liu M, Hu J, Lin F, et al. Filamentous GLS1 promotes ROS-induced apoptosis upon glutamine deprivation via insufficient asparagine synthesis. Mol Cell. 2022;82:1821–35.e6. 10.1016/j.molcel.2022.03.016 35381197

[12] Xue Y, Lu F, Chang Z, Li J, Gao Y, Zhou J, et al. Intermittent dietary methionine deprivation facilitates tumoral ferroptosis and synergizes with checkpoint blockade. Nat Commun. 2023;14:4758. 37553341 10.1038/s41467-023-40518-0 PMC10409767

[13] Liu X, Olszewski K, Zhang Y, Lim EW, Shi J, Zhang X, et al. Cystine transporter regulation of pentose phosphate pathway dependency and disulfide stress exposes a targetable metabolic vulnerability in cancer. Nat Cell Biol. 2020;22:476–86. 32231310 10.1038/s41556-020-0496-x PMC7194135

[14] Zhang Y, Shi J, Liu X, Feng L, Gong Z, Koppula P, et al. BAP1 links metabolic regulation of ferroptosis to tumour suppression. Nat Cell Biol. 2018;20:1181–92. 30202049 10.1038/s41556-018-0178-0 PMC6170713

[15] Dixon SJ, Lemberg KM, Lamprecht MR, Skouta R, Zaitsev EM, Gleason CE, et al. Ferroptosis: an iron-dependent form of nonapoptotic cell death. Cell. 2012;149:1060–72. 22632970 10.1016/j.cell.2012.03.042 PMC3367386

[16] Chen X, Li J, Kang R, Klionsky DJ, Tang D. Ferroptosis: machinery and regulation. Autophagy. 2021;17:2054–81. 32804006 10.1080/15548627.2020.1810918 PMC8496712

[17] Doll S, Proneth B, Tyurina YY, Panzilius E, Kobayashi S, Ingold I, et al. ACSL4 dictates ferroptosis sensitivity by shaping cellular lipid composition. Nat Chem Biol. 2017;13:91–8. 27842070 10.1038/nchembio.2239 PMC5610546

[18] Guo J, Xu B, Han Q, Zhou H, Xia Y, Gong C, et al. Ferroptosis: a novel anti-tumor action for cisplatin. Cancer Res Treat. 2018;50:445–60. 28494534 10.4143/crt.2016.572 PMC5912137

[19] Lang X, Green MD, Wang W, Yu J, Choi JE, Jiang L, et al. Radiotherapy and immunotherapy promote tumoral lipid oxidation and ferroptosis via synergistic repression of SLC7A11. Cancer Discov. 2019;9:1673–85. 31554642 10.1158/2159-8290.CD-19-0338 PMC6891128

[20] Wang W, Green M, Choi JE, Gijón M, Kennedy PD, Johnson JK, et al. CD8+ T cells regulate tumour ferroptosis during cancer immunotherapy. Nature. 2019;569:270–4. 31043744 10.1038/s41586-019-1170-y PMC6533917

[21] Masclef L, Ahmed O, Estavoyer B, Larrivée B, Labrecque N, Nijnik A, et al. Roles and mechanisms of BAP1 deubiquitinase in tumor suppression. Cell Death Differ. 2021;28:606–25. 33462414 10.1038/s41418-020-00709-4 PMC7862696

[22] Kundu S, Ji F, Sunwoo H, Jain G, Lee JT, Sadreyev RI, et al. Polycomb repressive complex 1 generates discrete compacted domains that change during differentiation. Mol Cell. 2017;65:432–46.e5. 10.1016/j.molcel.2017.01.009 PMC5421375 28157505

[23] Francis NJ, Kingston RE, Woodcock CL. Chromatin compaction by a polycomb group protein complex. Science. 2004;306:1574–7. 15567868 10.1126/science.1100576

[24] Shang Y, Luo M, Yao F, Wang S, Yuan Z, Yang Y. Ceruloplasmin suppresses ferroptosis by regulating iron homeostasis in hepatocellular carcinoma cells. Cell Signal. 2020;72:109633. 32283255 10.1016/j.cellsig.2020.109633

[25] Liao P, Wang W, Wang W, Kryczek I, Li X, Bian Y, et al. CD8+ T cells and fatty acids orchestrate tumor ferroptosis and immunity via ACSL4. Cancer Cell. 2022;40:365–78.e6. 10.1016/j.ccell.2022.02.003 PMC9007863 35216678

[26] Li J, Yuan J, Li Y, Wang J, Xie Q, Ma R, et al. d-Borneol enhances cisplatin sensitivity via autophagy dependent EMT signaling and NCOA4-mediated ferritinophagy. Phytomedicine. 2022;106:154411. 36030746 10.1016/j.phymed.2022.154411

[27] Chen F, Kang R, Liu J, Tang D. The ACSL4 network regulates cell death and autophagy in diseases. Biology. 2023;12:864. 37372148 10.3390/biology12060864 PMC10295397

[28] Bononi A, Wang Q, Zolondick AA, Bai F, Steele-Tanji M, Suarez JS, et al. BAP1 is a novel regulator of HIF-1α. Proc Natl Acad Sci USA. 2023;120:e2217840120. 36656861 10.1073/pnas.2217840120 PMC9942908

[29] Tao L, Yang X, Ge C, Zhang P, He W, Xu X, et al. Integrative clinical and preclinical studies identify FerroTerminator1 as a potent therapeutic drug for MASH. Cell Metab. 2024;36:2190–206.e5. 39142286 10.1016/j.cmet.2024.07.013

[30] Gao L, Zhang J, Yang T, Jiang L, Liu X, Wang S, et al. STING/ACSL4 axis-dependent ferroptosis and inflammation promote hypertension-associated chronic kidney disease. Mol Ther. 2023;31:3084–103. 37533255 10.1016/j.ymthe.2023.07.026 PMC10556226

[31] Qiu B, Zandkarimi F, Bezjian CT, Reznik E, Soni RK, Gu W, et al. Phospholipids with two polyunsaturated fatty acyl tails promote ferroptosis. Cell. 2024;187:1177–90.e18. 38366593 10.1016/j.cell.2024.01.030 PMC10940216

[32] Louie BH, Kurzrock R. BAP1: not just a BRCA1-associated protein. Cancer Treat Rev. 2020;90:102091. 32877777 10.1016/j.ctrv.2020.102091 PMC7655689

[33] Okino Y, Machida Y, Frankland-Searby S, Machida YJ. BRCA1-associated protein 1 (BAP1) deubiquitinase antagonizes the ubiquitin-mediated activation of FoxK2 target genes. J Biol Chem. 2015;290:1580–91. 25451922 10.1074/jbc.M114.609834 PMC4340404

[34] Harbour JW, Onken MD, Roberson EDO, Duan S, Cao L, Worley LA, et al. Frequent mutation of BAP1 in metastasizing uveal melanomas. Science. 2010;330:1410–3. 21051595 10.1126/science.1194472 PMC3087380

[35] Ye P, Mimura J, Okada T, Sato H, Liu T, Maruyama A, et al. Nrf2- and ATF4-dependent upregulation of xCT modulates the sensitivity of T24 bladder carcinoma cells to proteasome inhibition. Mol Cell Biol. 2014;34:3421–34. 25002527 10.1128/MCB.00221-14 PMC4135628

[36] Pascual G, Avgustinova A, Mejetta S, Martín M, Castellanos A, Attolini CS-O, et al. Targeting metastasis-initiating cells through the fatty acid receptor CD36. Nature. 2017;541:41–5. 27974793 10.1038/nature20791

[37] Calvisi DF, Wang C, Ho C, Ladu S, Lee SA, Mattu S, et al. Increased lipogenesis, induced by AKT-mTORC1-RPS6 signaling, promotes development of human hepatocellular carcinoma. Gastroenterology. 2011;140:1071–83. 21147110 10.1053/j.gastro.2010.12.006 PMC3057329

[38] Chen Y, Liang R, Li Y, Jiang L, Ma D, Luo Q, et al. Chromatin accessibility: biological functions, molecular mechanisms and therapeutic application. Signal Transduct Target Ther. 2024;9:340. 39627201 10.1038/s41392-024-02030-9 PMC11615378

[39] Bannister AJ, Kouzarides T. Regulation of chromatin by histone modifications. Cell Res. 2011;21:381–95. 21321607 10.1038/cr.2011.22 PMC3193420

[40] Wang H, Wang L, Erdjument-Bromage H, Vidal M, Tempst P, Jones RS, et al. Role of histone H2A ubiquitination in Polycomb silencing. Nature. 2004;431:873–8. 15386022 10.1038/nature02985

[41] Yin X, Romero-Campero FJ, de Los Reyes P, Yan P, Yang J, Tian G, et al. H2AK121ub in Arabidopsis associates with a less accessible chromatin state at transcriptional regulation hotspots. Nat Commun. 2021;12:315. 33436613 10.1038/s41467-020-20614-1 PMC7804394

[42] Artegiani B, van Voorthuijsen L, Lindeboom RGH, Seinstra D, Heo I, Tapia P, et al. Probing the tumor suppressor function of BAP1 in CRISPR-engineered human liver organoids. Cell Stem Cell. 2019;24:927–43.e6. 31130514 10.1016/j.stem.2019.04.017

[43] Röhrig F, Schulze A. The multifaceted roles of fatty acid synthesis in cancer. Nat Rev Cancer. 2016;16:732–49. 27658529 10.1038/nrc.2016.89

[44] Bian X, Liu R, Meng Y, Xing D, Xu D, Lu Z. Lipid metabolism and cancer. J Exp Med. 2021;218:e20201606. 33601415 10.1084/jem.20201606 PMC7754673

[45] Grube J, Woitok MM, Mohs A, Erschfeld S, Lynen C, Trautwein C, et al. ACSL4-dependent ferroptosis does not represent a tumor-suppressive mechanism but ACSL4 rather promotes liver cancer progression. Cell Death Dis. 2022;13:704. 35963845 10.1038/s41419-022-05137-5 PMC9376109

[46] Dang W, Li Q, Wang X. ACSL4 promotes the formation of the proliferative subtype in hepatoblastoma. BMC Cancer. 2025;25:191. 39901207 10.1186/s12885-025-13592-4 PMC11789379

[47] Wang Y, Hu M, Cao J, Wang F, Han JR, Wu TW, et al. ACSL4 and polyunsaturated lipids support metastatic extravasation and colonization. Cell. 2025;188:412–29.e27. 39591965 10.1016/j.cell.2024.10.047

[48] Sinha A, Saini KK, Chandramouli A, Tripathi K, Khan MA, Satrusal SR, et al. ACSL4-mediated H3K9 and H3K27 hyperacetylation upregulates SNAIL to drive TNBC metastasis. Proc Natl Acad Sci USA. 2024;121:e2408049121. 39700137 10.1073/pnas.2408049121 PMC11670210

[49] Qiu Y, Wang X, Sun Y, Jin T, Tang R, Zhou X, et al. ACSL4-mediated membrane phospholipid remodeling induces integrin β1 activation to facilitate triple-negative breast cancer metastasis. Cancer Res. 2024;84:1856–71. 38471082 10.1158/0008-5472.CAN-23-2491 PMC11148537

[50] Qin J, Zhou Z, Chen W, Wang C, Zhang H, Ge G, et al. BAP1 promotes breast cancer cell proliferation and metastasis by deubiquitinating KLF5. Nat Commun. 2015;6:8471. 26419610 10.1038/ncomms9471 PMC4598844

[51] Lee J, Roh J-L. Lipid metabolism in ferroptosis: unraveling key mechanisms and therapeutic potential in cancer. Biochim Biophys Acta Rev Cancer. 2025;1880:189258. 39746458 10.1016/j.bbcan.2024.189258

[52] Bai Y, Meng L, Han L, Jia Y, Zhao Y, Gao H, et al. Lipid storage and lipophagy regulates ferroptosis. Biochem Biophys Res Commun. 2019;508:997–1003. 30545638 10.1016/j.bbrc.2018.12.039

[53] Wang J, Wang M, Wu S, Zhu Y, Fan K, Chen Y, et al. Tumor suppressor BAP1 suppresses disulfidptosis through the regulation of SLC7A11 and NADPH levels. Oncogenesis. 2024;13:31. 39266549 10.1038/s41389-024-00535-0 PMC11393423

[54] Yang WS, SriRamaratnam R, Welsch ME, Shimada K, Skouta R, Viswanathan VS, et al. Regulation of ferroptotic cancer cell death by GPX4. Cell. 2014;156:317–31. 24439385 10.1016/j.cell.2013.12.010 PMC4076414

[55] Kechin A, Boyarskikh U, Kel A, Filipenko M. cutPrimers: a new tool for accurate cutting of primers from reads of targeted next generation sequencing. J Comput Biol. 2017;24:1138–43. 28715235 10.1089/cmb.2017.0096

[56] Langmead B, Salzberg SL. Fast gapped-read alignment with Bowtie 2. Nat Methods. 2012;9:357–9. 22388286 10.1038/nmeth.1923 PMC3322381

[57] Danecek P, Bonfield JK, Liddle J, Marshall J, Ohan V, Pollard MO, et al. Twelve years of SAMtools and BCFtools. Gigascience. 2021;10:giab008. 33590861 10.1093/gigascience/giab008 PMC7931819

[58] Zhang Y, Liu T, Meyer CA, Eeckhoute J, Johnson DS, Bernstein BE, et al. Model-based analysis of ChIP-Seq (MACS). Genome Biol. 2008;9:R137. 18798982 10.1186/gb-2008-9-9-r137 PMC2592715

[59] Yu G, Wang L-G, He Q-Y. ChIPseeker: an R/Bioconductor package for ChIP peak annotation, comparison and visualization. Bioinformatics. 2015;31:2382–3. 25765347 10.1093/bioinformatics/btv145

[60] Chen T, Chen X, Zhang S, Zhu J, Tang B, Wang A, et al. The genome sequence archive family: toward explosive data growth and diverse data types. Genom Proteom Bioinform. 2021;19:578–83. 10.1016/j.gpb.2021.08.001 PMC9039563 34400360

[61] CNCB-NGDC Members and Partners. Database Resources of the National Genomics Data Center, China National Center for Bioinformation in 2022. Nucleic Acids Res. 2022;50:D27-38. 10.1093/nar/gkab951 PMC8728233 34718731

