# Supplementary material for: Epigenetic regulation of ACSL4 via H2A monoubiquitylation connects lipid metabolism to BAP1-mediated ferroptosis
Source: Cell Death Differ. 2025 Nov 27;33(6):1136–51. doi: 10.1038/s41418-025-01624-2 (PMC13246907; doi:10.1038/s41418-025-01624-2)
Supplement: Supplementary file 1 — Supplementary information [file 41418_2025_1624_MOESM1_ESM.pdf]

Supplementary Information

Epigenetic Regulation of ACSL4 via H2A Monoubiquitylation Connects Lipid Metabolism to BAP1-Mediated Ferroptosis

Running title: BAP1 Regulates Lipid Metabolism and Ferroptosis through ACSL4

Kexin Fan[1]*, Shuting Zhou[2]*, Yakun Ren[1], Jingwen Xiong[1], Hua Wang[1], Yaxin Fu[1], Yuhan Chen[1], Bobo Wang[3], Kun Fan[1], Min Gao[1], Tingli Guo[1], Xiaofeng Wei[3], Lianying Jiao[1], Jiejun Shi[2#], Chenguang Ding[1#], Yilei Zhang[1#]

1Department of Biochemistry and Molecular Biology, School of Basic Medical Sciences, Department of Kidney Transplantation, Department of Organ Procurement and Allocation, The First Affiliated Hospital, Xi'an Jiaotong University, Xi'an, Shaanxi 710061, China;

2 Key Laboratory of Spine and Spinal Cord Injury Repair and Regeneration of Ministry of Education, Tongji Hospital affiliated to Tongji University, Frontier Science Center for Stem Cell Research, School of Life Sciences and Technology, Tongji University, Shanghai, 200092, China;

3 School of Pharmacy, Xi'an Jiaotong University, Xi'an, Shaanxi 710061, China;

*These authors contributed equally to this work

[#]Correspondence to:

Yilei Zhang (zhangyilei@xjtu.edu.cn)

Chenguang Ding (doctor_ding@xjtu.edu.cn)

Jiejun Shi (shij@tongji.edu.cn)

Fig. S1 BAP1 regulates target genes transcription and metabolism-related biological processes through chromatin accessibility in an H2Aub-dependent manner

A. MA plot of log2-fold changes of the corresponding genes expression levels in EV and BAP1 WT cells. **B-C.** GO biological process and KEGG analysis for the 983 upregulated (**B**) and 717 downregulated (**C**) genes. Annotation clusters (in part) are shown according to their enrichment scores [−log10 (*P*-value)]. **D-E.** GSEA revealed that genes associated with ferroptosis (**D**) and fatty acid biosynthesis (**E**) are significantly enriched in gene expression differences between EV and BAP1 WT cells. **F.** MA plot of log2-fold changes of the H2Aub occupancies levels in EV and BAP1 WT cells. **G.** Schematic workflow for identifying relevant BAP1 target genes with H2Aub-dependent chromatin remodeling in cancer.

Fig. S2 BAP1 promotes ferroptosis independently of its regulation of SLC7A11

A. The Sankey diagram visualizes that the 91 genes with > 1.6-fold H2Aub reduction, > 1.5-fold chromatin opening and > 1.5-fold gene expression increased were enriched in the corresponding biological metabolic pathway (the analysis reveals only a subset of pathways). **B.** Cell viability measured by CCK-8 assay in indicated cell lines cultured in medium with different concentrations of erastin. **C.** Representative fluorescence intensity images of cellular lipid ROS by BODIPY 581/591 C11 obtained by flow cytometry. **D.** Cell viability measured by CCK-8 assay in indicated cell lines cultured in medium with different concentrations of IKE. **E.** Representative fluorescence intensity images of cellular lipid ROS by BODIPY 581/591 C11 obtained by flow cytometry with the illustrated treatment. **F.** Cell viability measured by CCK-8 assay in indicated cell lines cultured in medium with different concentrations of cystine. **G.** Representative fluorescence intensity images of cellular lipid

ROS by BODIPY 581/591 C11 obtained by flow cytometry with the illustrated treatment. **H.** Western blotting analysis of the indicated protein expression in NCI-H226 cell lines. *n* = 3. **I.** Cell viability measured by CCK-8 assay in SLC7A11 KO-EV, -BAP1, -C91A NCI-H226 cell lines cultured in medium with different concentrations of IKE. **J.** Cell viability measured by CCK-8 assay in indicated NCI-H226 cells cultured in cystine-containing/-free medium. Error bars are mean ± SD. All *P*-values were calculated using two-tailed unpaired Student's t-test. *n* ≥ 3 independent repeats unless specified. ns: not significant (*P* > 0.05).

Fig. S3 BAP1 upregulates ACSL4 expression and reduces H2Aub occupancy on the ACSL4 promoter

A-C. mRNA levels of *CP* (**A**), *PRNP* (**B**) and *SLC7A11* (**C**) in indicated UMRC6 cells were measured by RT-PCR. **D-E.** Protein levels of indicated genes in BAP1 OE (**D**) and BAP1 KO (**E**) MDA-MB-231 cells were measured by Western blotting. *n* = 3. **F.** UMRC6 -EV, -BAP1 cells were treated with CHX for the indicated time with or without 10 μM MG132 followed by Western blotting analysis. **G.** Summary scheme of the upstream regulatory factors of ACSL4 and their inhibitors. **H-J.** mRNA levels of *HIF-1α* (**H**), *MYC* (**I**) and *STING* (**J**) in indicated UMRC6 cells were measured by RT-PCR. **K-M.** Western blotting analysis of indicated proteins expression in HIF-1α KO (**K**), c-Myc KO (**L**) and STING KO (**M**) related cell lines. *n* = 3. **N.** The scatter plots show the positive correlation of BAP1 and ACSL4 expression in other different cell lines from different tissues. Error bars are mean ± SD. All *P*-values were calculated using two-tailed unpaired Student's t-test. *n* ≥ 3 independent repeats unless specified. ns: not significant (*P* > 0.05).

Fig. S4 BAP1 regulates ferroptosis through ACSL4

A. Cell growth assays of 786-O cells with indicated genotypes. **B.** Representative fluorescence intensity images of cellular lipid ROS by BODIPY 581/591 C11 obtained by flow cytometry with the illustrated treatment. **C.** Cell viability measured by CCK-8 assay in indicated cell lines cultured in medium with different concentrations of ML162. **D.** Bar graph showing cell viability in indicated cells treated with ML162 (0.2 μM) combined with the inhibitors. **E.** Western blotting analysis of ACSL4 expression in ACSL4 KO UMRC6 cells. *n* = 5. **F.** Cell viability measured by CCK-8 assay in indicated cell lines treated with different concentrations of erastin. **G.** Bar graph showing cell viability in indicated cells treated with erastin (10 μM) combined with different inhibitors. **H-I.** Bar graph showing cell viability in indicated cells treated with cystine limitation (**H**) and ferroptosis inhibitors (**I**). **J.** Cell viability measured by CCK-8 assay in indicated cell lines cultured in medium with different concentrations of RSL3. **K.** Cell growth assays of UMRC6 cells with indicated genotypes. **L.** Representative fluorescence intensity images of cellular lipid ROS by BODIPY 581/591 C11 obtained by flow cytometry. **M-O.** Bar graph showing cell viability in indicated cells treated with TBH (10 μM) combined with HIF-1α inhibitor PX-478 (1 μM) (**M**), c-Myc inhibitor 10058-F4 (2 μM) (**N**) or STING inhibitor SN-011 (1 μM) (**O**). Error bars are mean ± SD. All *P*-values were calculated using two-tailed unpaired Student's t-test. $n \geq 3$ independent repeats unless specified. ns: not significant ($P > 0.05$).

Fig. S5 ACSL4-mediated lipid metabolism and ferroptosis sensitivity regulated by BAP1

A-C. Relative lipid abundance of TG (**A**), PC (**B**) and PE (**C**) in UMRC6-EV and -BAP1 cells. **D.** Diagram showing the structure of PL-$PUFA_{1s}$ and PL-$PUFA_{2s}$. **E.** Cell morphological changes in indicated cells were measured after treatment with TBH combined with AA or AA-CoA for 6 h. Error bars are mean ± SD. All *P*-values were calculated using two-tailed unpaired Student's t-test. $n \geq 3$ independent

repeats unless specified. ns: not significant ($P > 0.05$).

Fig. S6 ASXLs are required for the regulation of gene expression and ferroptosis by BAP1

A-B. Co-immunoprecipitation and Western blotting analysis of interactions between BAP1 and ASXL1 (**A**), ASXL2 (**B**) in HEK293T cells transfected with the specified plasmids. $n = 3$. **C.** Phase-contrast images show the cell morphological changes in the indicated cells after treatment with 10 μM erastin for 24 h. **D.** Representative fluorescence intensity images of cellular lipid ROS by BODIPY 581/591 C11 obtained by flow cytometry in the indicated cells after treatment with 10 μM erastin for 24 h. **E-F.** Cell growth assays of UMRC6 cells with indicated genotypes. **G-H.** Protein levels of indicated genes in ASXL1 KO (**G**) and ASXL2 KO (**H**) UMRC6 cells were measured by Western blotting. $n = 3$. **I.** Analysis of The Cancer Therapeutics Response Portal (CTRP) database reveals an association between ASXL1/ASXL2 expression and differential sensitivity to ferroptosis inducers (erastin, ML162, RSL3, ML210). **J-K.** Cell viability in indicated cells was measured after treatment with different concentrations of erastin in ASXL1 KO (**J**) and ASXL2 KO (**K**) cells. **L.** Western blotting analysis of ACSL4 expression in ASXL2 KO UMRC6 cell restored with BAP1. $n = 3$. **M.** Cell viability in indicated cells was measured after treatment with different concentrations of IKE. **N.** The bar graph shows the cell viability in indicated cells under cystine-limited conditions. Error bars are mean ± SD. All *P*-values were calculated using two-tailed unpaired Student's t-test. $n \geq 3$ independent repeats unless specified. ns: not significant ($P > 0.05$).

Fig. S1

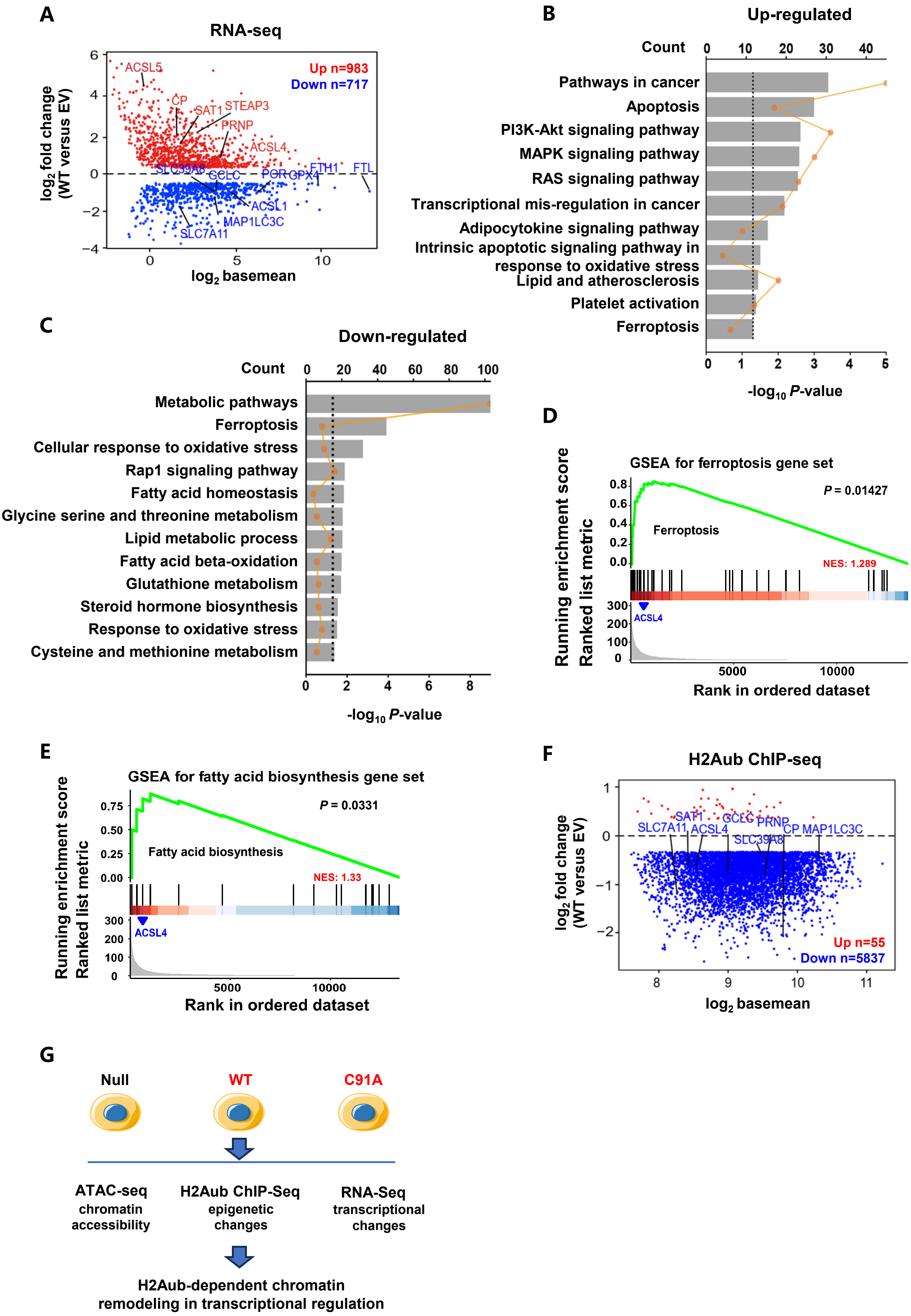

A
RNA-seq
Up n=983
Down n=717
log2 fold change (WT versus EV)
log2 basemean
ACSL5
CP
SAT1
STEAP3
PRNP
ACSL4
SLC39A8
GCLC
POR
GPX4
FTH1
FTL
ACSL1
MAP1LC3C
SLC7A11
B
Up-regulated
Count
Pathways in cancer
Apoptosis
PI3K-Akt signaling pathway
MAPK signaling pathway
RAS signaling pathway
Transcriptional mis-regulation in cancer
Adipocytokine signaling pathway
Intrinsic apoptotic signaling pathway in response to oxidative stress
Lipid and atherosclerosis
Platelet activation
Ferroptosis
-log10 P-value
C
Down-regulated
Count
Metabolic pathways
Ferroptosis
Cellular response to oxidative stress
Rap1 signaling pathway
Fatty acid homeostasis
Glycine serine and threonine metabolism
Lipid metabolic process
Fatty acid beta-oxidation
Glutathione metabolism
Steroid hormone biosynthesis
Response to oxidative stress
Cysteine and methionine metabolism
-log10 P-value
D
GSEA for ferroptosis gene set
P = 0.01427
Ferroptosis
NES: 1.289
ACSL4
Running enrichment score
Ranked list metric
Rank in ordered dataset
E
GSEA for fatty acid biosynthesis gene set
P = 0.0331
Fatty acid biosynthesis
NES: 1.33
ACSL4
Running enrichment score
Ranked list metric
Rank in ordered dataset
F
H2Aub ChIP-seq
SAT1
GCLC
PRNP
SLC7A11
ACSL4
CP
MAP1LC3C
SLC39A8
Up n=55
Down n=5837
log2 fold change (WT versus EV)
log2 basemean
G
Null
WT
C91A
ATAC-seq
chromatin accessibility
H2Aub ChIP-Seq
epigenetic changes
RNA-Seq
transcriptional changes
H2Aub-dependent chromatin remodeling in transcriptional regulation

Fig. S2

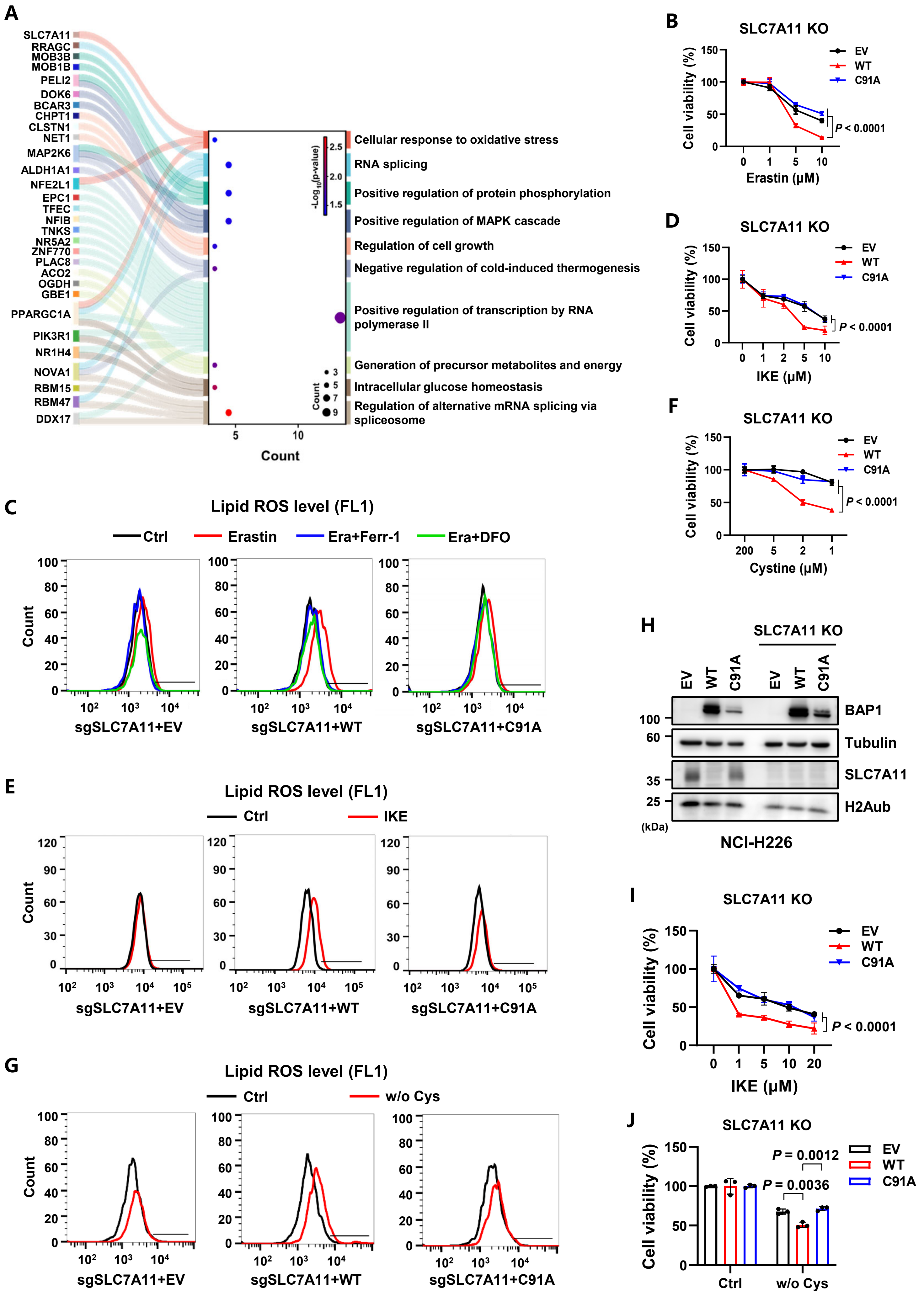

A
SLC7A11
RRAGC
MOB3B
MOB1B
PELI2
DOK6
BCAR3
CHPT1
CLSTN1
NET1
MAP2K6
ALDH1A1
NFE2L1
EPC1
TFEC
NFIB
TNKS
NR5A2
ZNF770
PLAC8
ACO2
OGDH
GBE1
PPARGC1A
PIK3R1
NR1H4
NOVA1
RBM15
RBM47
DDX17
-Log10(p-value)
Count
Cellular response to oxidative stress
RNA splicing
Positive regulation of protein phosphorylation
Positive regulation of MAPK cascade
Regulation of cell growth
Negative regulation of cold-induced thermogenesis
Positive regulation of transcription by RNA polymerase II
Generation of precursor metabolites and energy
Intracellular glucose homeostasis
Regulation of alternative mRNA splicing via spliceosome
B
SLC7A11 KO
Cell viability (%)
Erastin (μM)
EV
WT
C91A
P < 0.0001
C
Lipid ROS level (FL1)
Ctrl
Erastin
Era+Ferr-1
Era+DFO
Count
sgSLC7A11+EV
sgSLC7A11+WT
sgSLC7A11+C91A
D
SLC7A11 KO
Cell viability (%)
IKE (μM)
P < 0.0001
E
Lipid ROS level (FL1)
Ctrl
IKE
F
SLC7A11 KO
Cystine (μM)
P < 0.0001
G
Lipid ROS level (FL1)
Ctrl
w/o Cys
H
SLC7A11 KO
EV
WT
C91A
BAP1
Tubulin
SLC7A11
H2Aub
(kDa)
NCI-H226
I
SLC7A11 KO
IKE (μM)
P < 0.0001
J
SLC7A11 KO
P = 0.0012
P = 0.0036
Ctrl
w/o Cys

Fig. S3

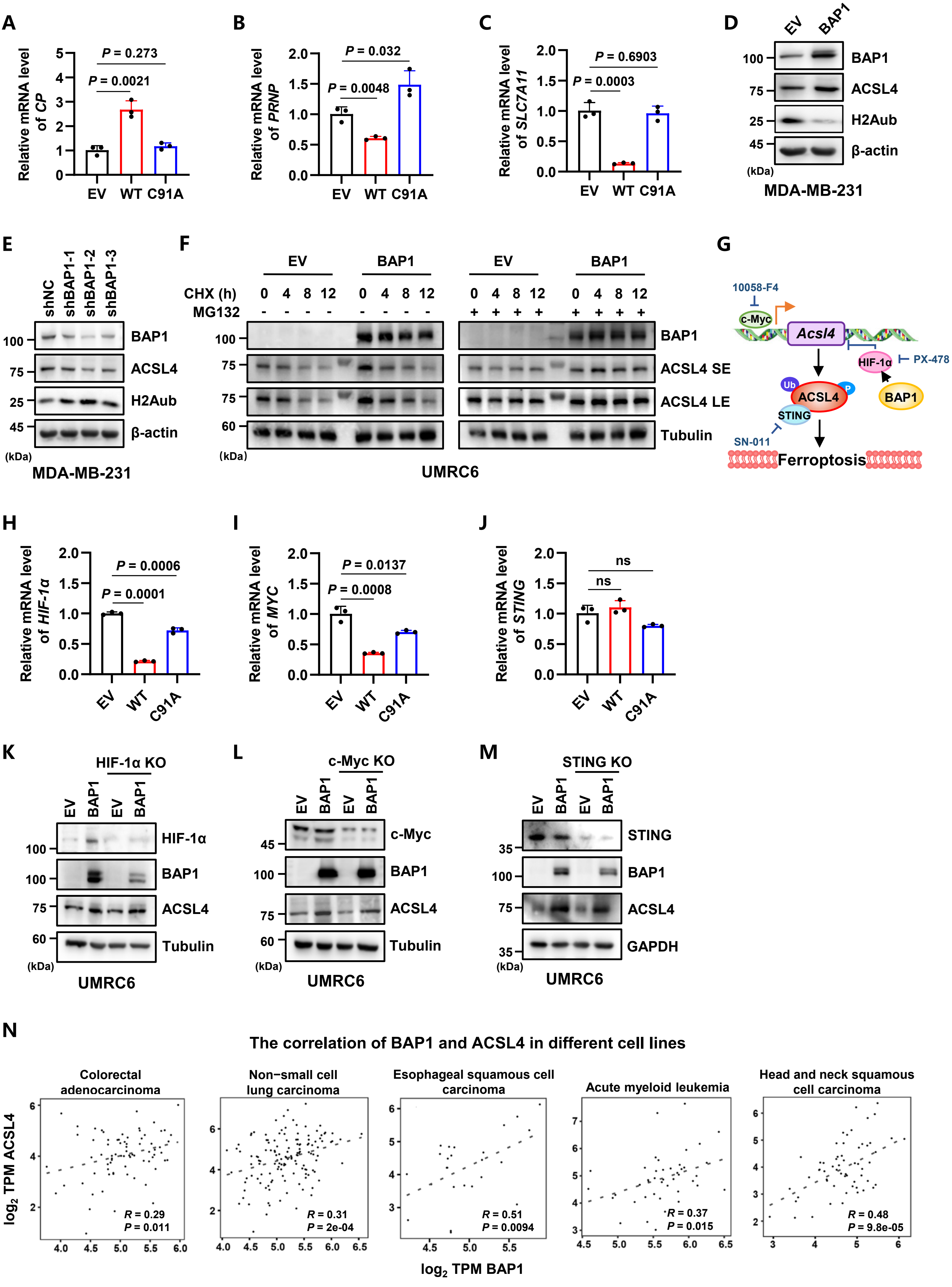

A
Relative mRNA level of CP
P = 0.273
P = 0.0021
EV
WT
C91A
B
Relative mRNA level of PRNP
P = 0.032
P = 0.0048
EV
WT
C91A
C
Relative mRNA level of SLC7A11
P = 0.6903
P = 0.0003
EV
WT
C91A
D
EV
BAP1
BAP1
ACSL4
H2Aub
β-actin
(kDa)
MDA-MB-231
E
shNC
shBAP1-1
shBAP1-2
shBAP1-3
BAP1
ACSL4
H2Aub
β-actin
(kDa)
MDA-MB-231
F
EV
BAP1
CHX (h)
MG132
BAP1
ACSL4 SE
ACSL4 LE
Tubulin
(kDa)
UMRC6
G
10058-F4
c-Myc
Acsl4
HIF-1α
PX-478
Ub
ACSL4
P
BAP1
STING
SN-011
Ferroptosis
H
Relative mRNA level of HIF-1α
P = 0.0006
P = 0.0001
EV
WT
C91A
I
Relative mRNA level of MYC
P = 0.0137
P = 0.0008
EV
WT
C91A
J
Relative mRNA level of STING
ns
ns
EV
WT
C91A
K
HIF-1α KO
EV
BAP1
HIF-1α
BAP1
ACSL4
Tubulin
(kDa)
UMRC6
L
c-Myc KO
EV
BAP1
c-Myc
BAP1
ACSL4
Tubulin
(kDa)
UMRC6
M
STING KO
EV
BAP1
STING
BAP1
ACSL4
GAPDH
(kDa)
UMRC6
N
The correlation of BAP1 and ACSL4 in different cell lines
Colorectal adenocarcinoma
R = 0.29
P = 0.011
Non−small cell lung carcinoma
R = 0.31
P = 2e-04
Esophageal squamous cell carcinoma
R = 0.51
P = 0.0094
Acute myeloid leukemia
R = 0.37
P = 0.015
Head and neck squamous cell carcinoma
R = 0.48
P = 9.8e-05
log2 TPM ACSL4
log2 TPM BAP1

Fig. S4

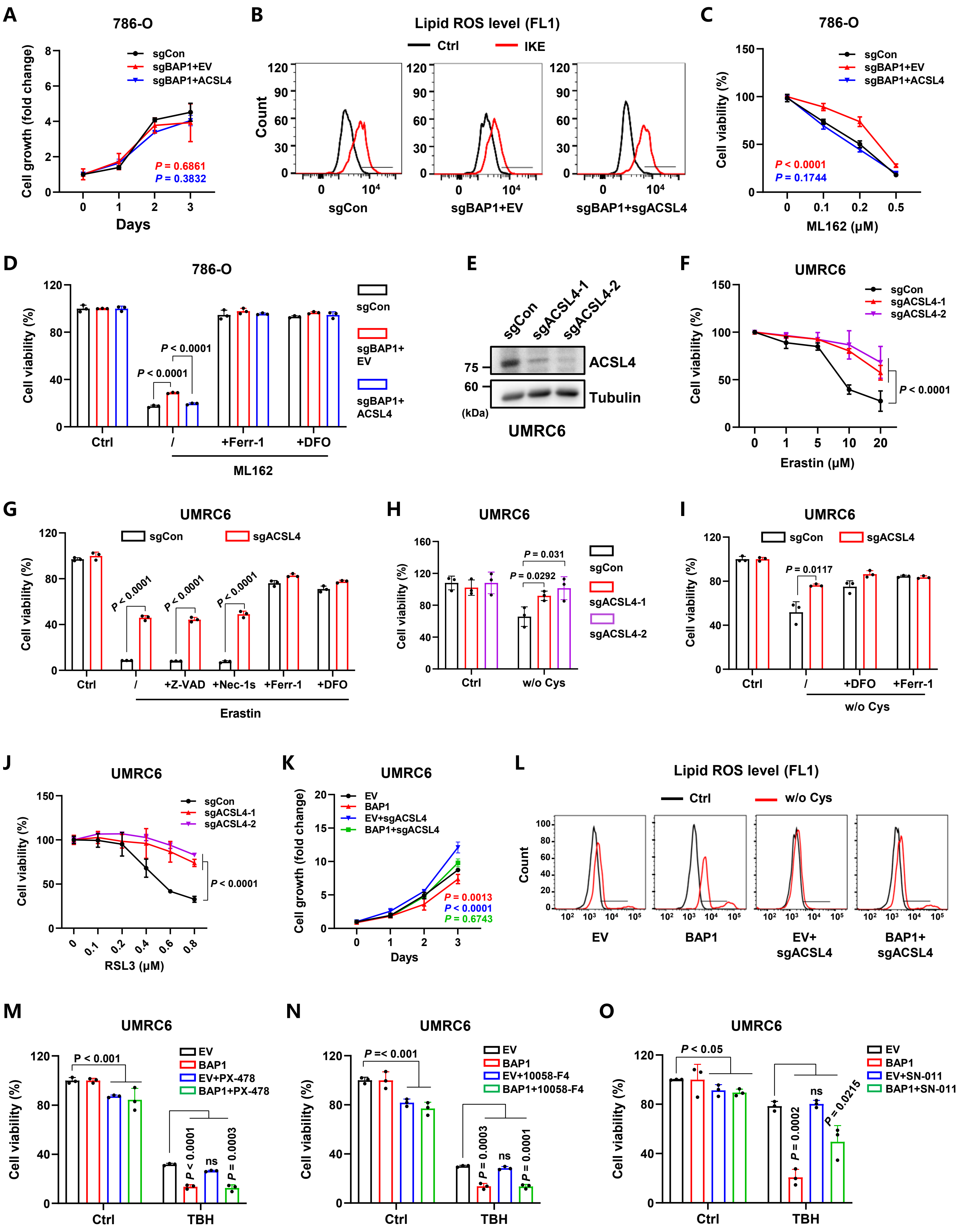
A
786-O
Cell growth (fold change)
Days
sgCon
sgBAP1+EV
sgBAP1+ACSL4
P = 0.6861
P = 0.3832
B
Lipid ROS level (FL1)
Ctrl
IKE
Count
sgCon
sgBAP1+EV
sgBAP1+sgACSL4
C
786-O
Cell viability (%)
ML162 (μM)
P < 0.0001
P = 0.1744
D
786-O
Ctrl
+Ferr-1
+DFO
ML162
P < 0.0001
P < 0.0001
sgBAP1+ EV
sgBAP1+ ACSL4
E
sgCon
sgACSL4-1
sgACSL4-2
ACSL4
Tubulin
(kDa)
UMRC6
F
UMRC6
Erastin (μM)
P < 0.0001
G
UMRC6
sgACSL4
+Z-VAD
+Nec-1s
Erastin
H
UMRC6
w/o Cys
P = 0.031
P = 0.0292
I
UMRC6
P = 0.0117
J
UMRC6
RSL3 (μM)
K
UMRC6
EV
BAP1
EV+sgACSL4
BAP1+sgACSL4
P = 0.0013
P < 0.0001
P = 0.6743
L
Lipid ROS level (FL1)
EV+ sgACSL4
BAP1+ sgACSL4
M
UMRC6
EV+PX-478
BAP1+PX-478
P < 0.001
TBH
P < 0.0001
ns
P = 0.0003
N
UMRC6
EV+10058-F4
BAP1+10058-F4
P =< 0.001
P = 0.0003
P = 0.0001
O
UMRC6
EV+SN-011
BAP1+SN-011
P < 0.05
P = 0.0002
P = 0.0215

Fig. S5

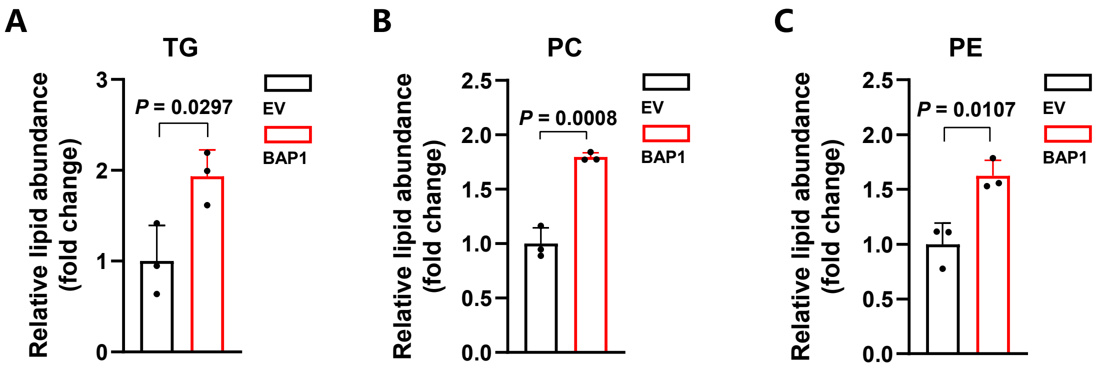
A
TG
Relative lipid abundance (fold change)
P = 0.0297
EV
BAP1
B
PC
P = 0.0008
C
PE
P = 0.0107

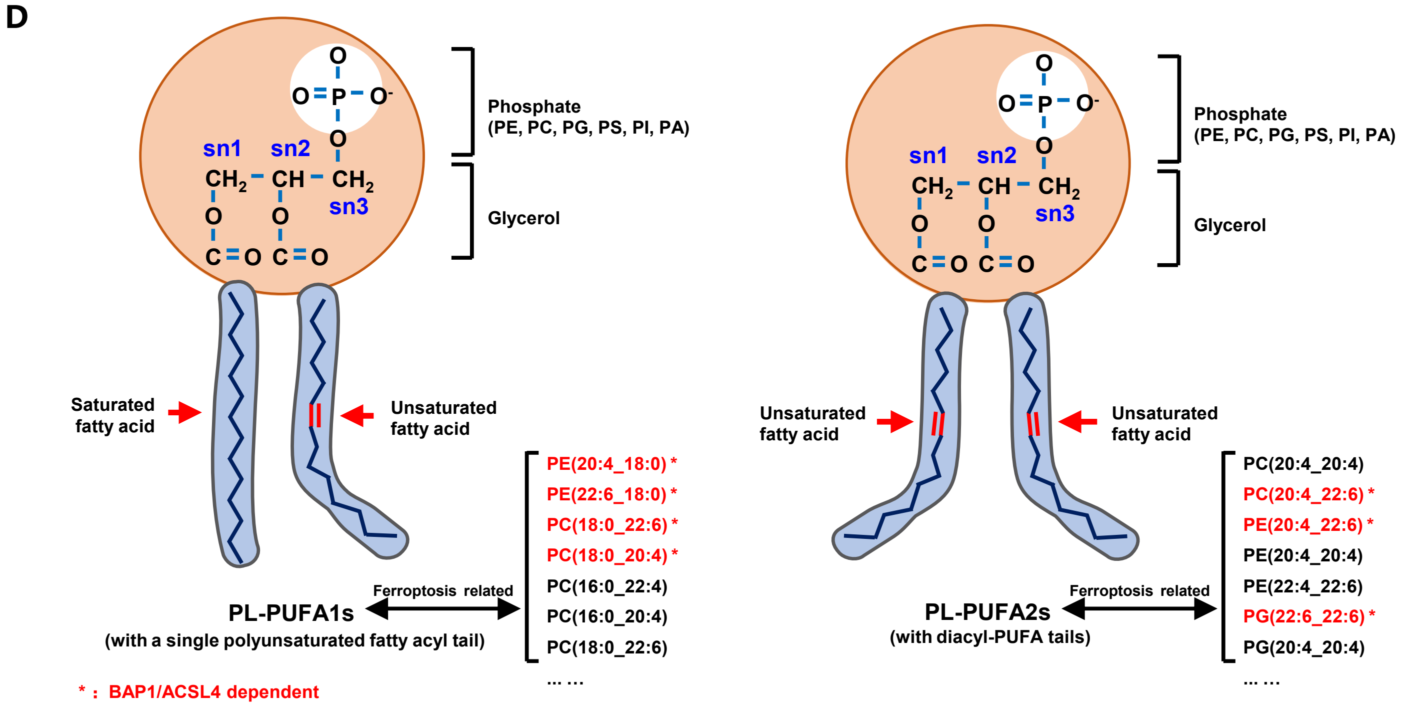
D
Phosphate
(PE, PC, PG, PS, PI, PA)
Glycerol
sn1
sn2
sn3
Saturated fatty acid
Unsaturated fatty acid
PL-PUFA1s
(with a single polyunsaturated fatty acyl tail)
Ferroptosis related
PE(20:4_18:0) *
PE(22:6_18:0) *
PC(18:0_22:6) *
PC(18:0_20:4) *
PC(16:0_22:4)
PC(16:0_20:4)
PC(18:0_22:6)
... ...
* : BAP1/ACSL4 dependent
PL-PUFA2s
(with diacyl-PUFA tails)
PC(20:4_20:4)
PC(20:4_22:6) *
PE(20:4_22:6) *
PE(20:4_20:4)
PE(22:4_22:6)
PG(22:6_22:6) *
PG(20:4_20:4)
... ...

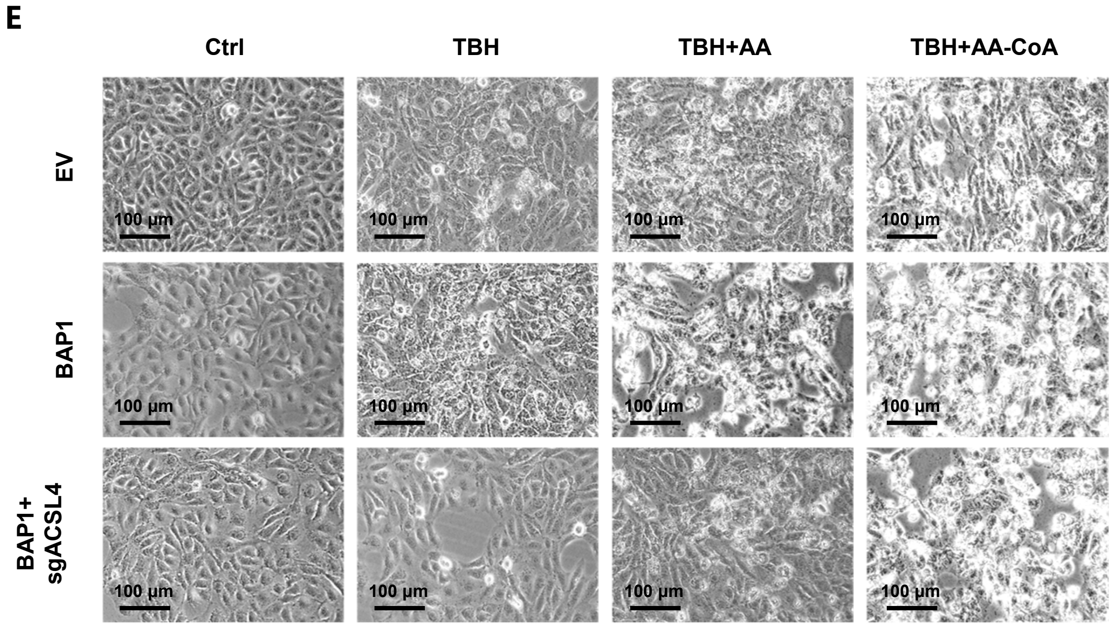
E
Ctrl
TBH
TBH+AA
TBH+AA-CoA
EV
BAP1
BAP1+ sgACSL4
100 μm

Fig. S6

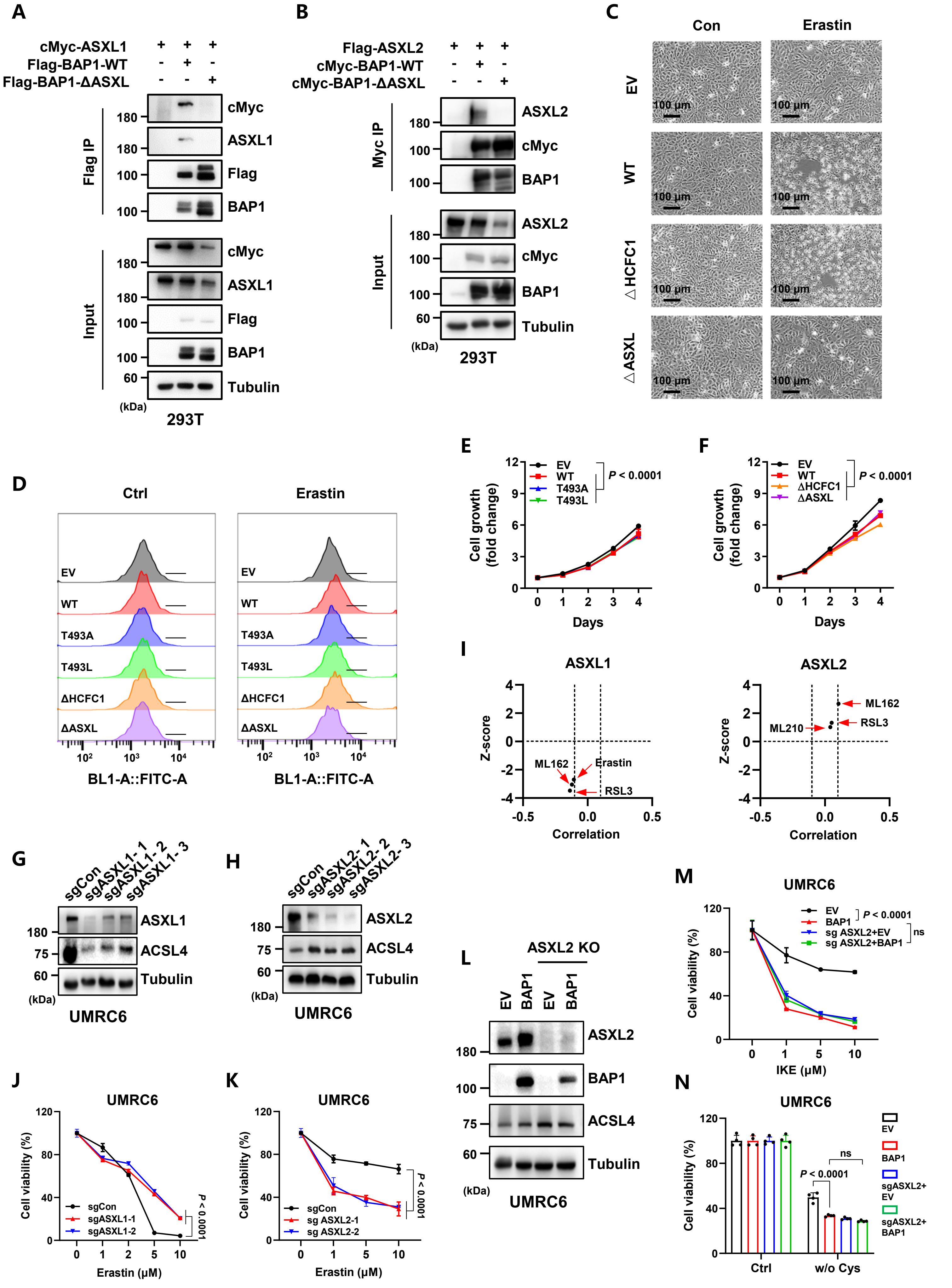

A
cMyc-ASXL1
Flag-BAP1-WT
Flag-BAP1-ΔASXL
Flag IP
Input
cMyc
ASXL1
Flag
BAP1
Tubulin
(kDa)
293T
B
Flag-ASXL2
cMyc-BAP1-WT
cMyc-BAP1-ΔASXL
Myc IP
Input
ASXL2
cMyc
BAP1
Tubulin
(kDa)
293T
C
Con
Erastin
EV
WT
△HCFC1
△ASXL
100 μm
D
Ctrl
Erastin
EV
WT
T493A
T493L
ΔHCFC1
ΔASXL
BL1-A::FITC-A
E
Cell growth (fold change)
Days
EV
WT
T493A
T493L
P < 0.0001
F
Cell growth (fold change)
Days
EV
WT
ΔHCFC1
ΔASXL
P < 0.0001
I
ASXL1
Z-score
Correlation
ML162
Erastin
RSL3
ASXL2
ML162
RSL3
ML210
G
sgCon
sgASXL1-1
sgASXL1-2
sgASXL1-3
ASXL1
ACSL4
Tubulin
(kDa)
UMRC6
H
sgCon
sgASXL2-1
sgASXL2-2
sgASXL2-3
ASXL2
ACSL4
Tubulin
(kDa)
UMRC6
L
ASXL2 KO
EV
BAP1
EV
BAP1
ASXL2
BAP1
ACSL4
Tubulin
(kDa)
UMRC6
M
UMRC6
Cell viability (%)
IKE (μM)
EV
BAP1
sg ASXL2+EV
sg ASXL2+BAP1
P < 0.0001
ns
J
UMRC6
Cell viability (%)
Erastin (μM)
sgCon
sgASXL1-1
sgASXL1-2
P < 0.0001
K
UMRC6
Cell viability (%)
Erastin (μM)
sgCon
sg ASXL2-1
sg ASXL2-2
P < 0.0001
N
UMRC6
Cell viability (%)
Ctrl
w/o Cys
EV
BAP1
sgASXL2+EV
sgASXL2+BAP1
P < 0.0001
ns
