# Supplementary material for: Epigenetic regulation of ACSL4 via H2A monoubiquitylation connects lipid metabolism to BAP1-mediated ferroptosis
Source: Cell Death Differ. 2025 Nov 27;33(6):1136–51. doi: 10.1038/s41418-025-01624-2 (PMC13246907; doi:10.1038/s41418-025-01624-2)
Supplement: Supplementary file 5 — Supplementary Material-uncropped western blots [file 41418_2025_1624_MOESM5_ESM.pdf]

Fig 1A

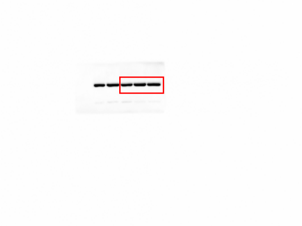

Tubulin

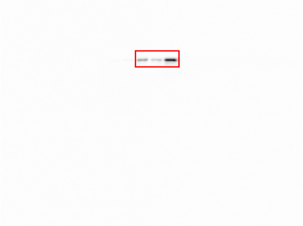

H2Aub

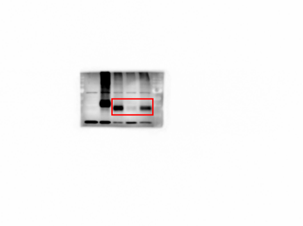

SLC7A11

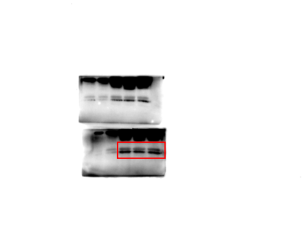

H2Aub

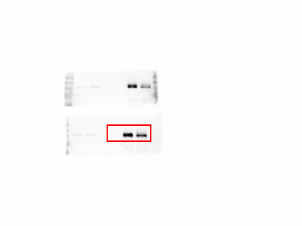

BAP1

Fig 2B

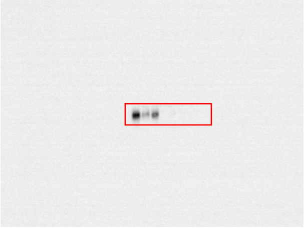

SLC7A11

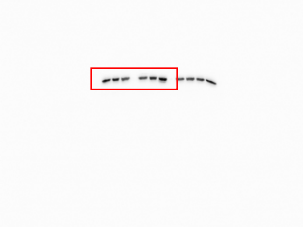

Tubulin

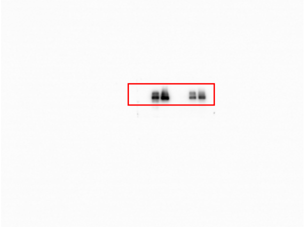

BAP1

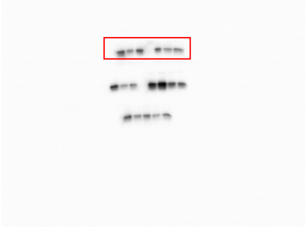

H2Aub

Fig S2H

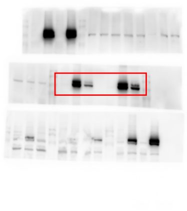

BAP1

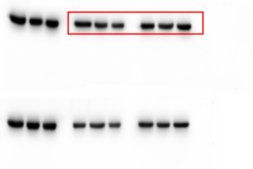

Tubulin

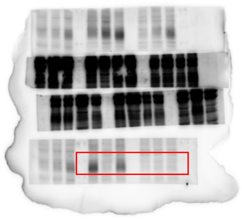

SLC7A11

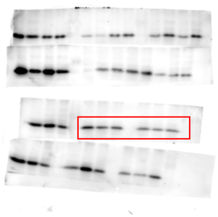

H2Aub

Fig 3B

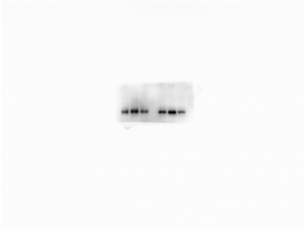
ACSL4

Tubulin

Fig 3D

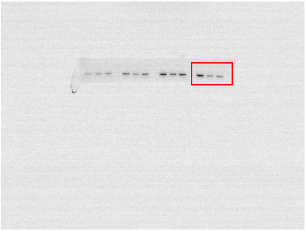
ACSL4

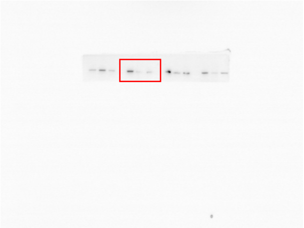
BAP1

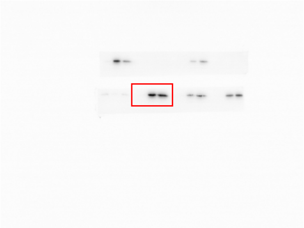
H2Aub

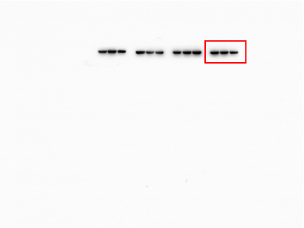
Tubulin

Fig 3H

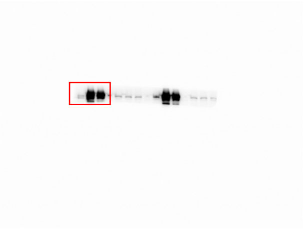
BAP1

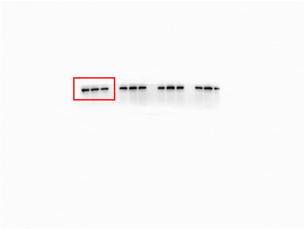
Tubulin

Fig 3I

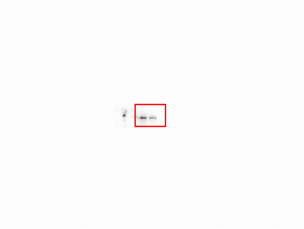
BAP1

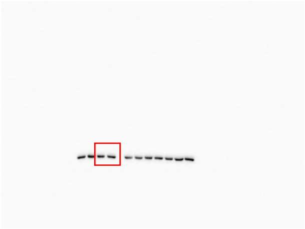
Tubulin

Fig 3K

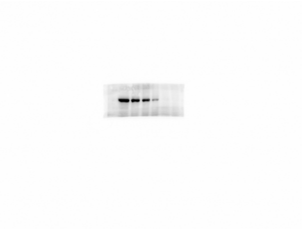
BAP1

ACSL4

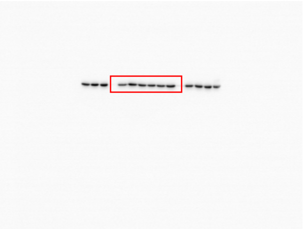
Tubulin

Fig S3D, E

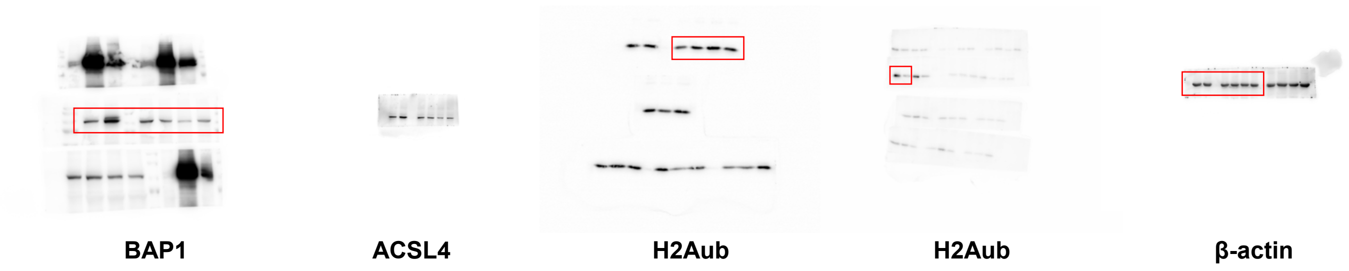

Fig S3F

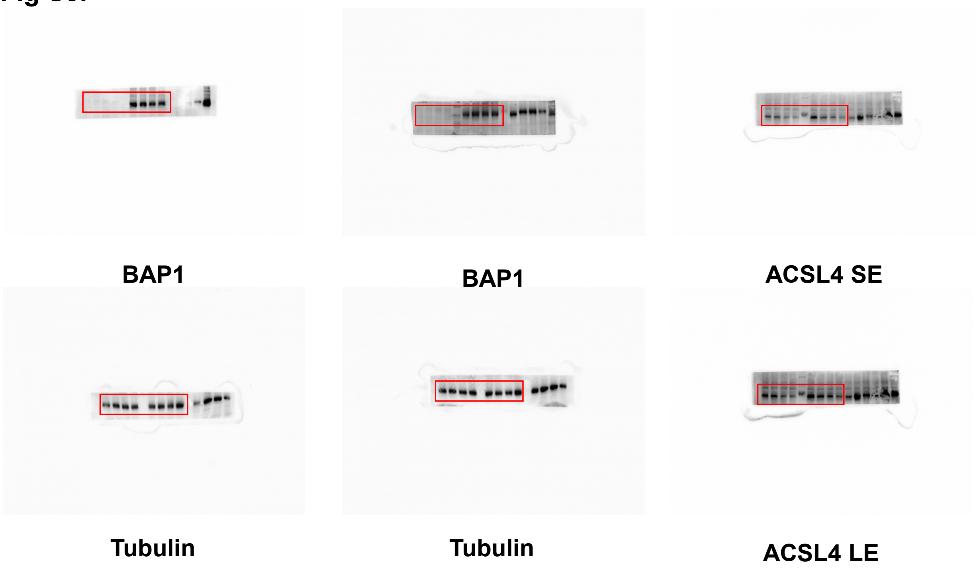

Fig S3K-M

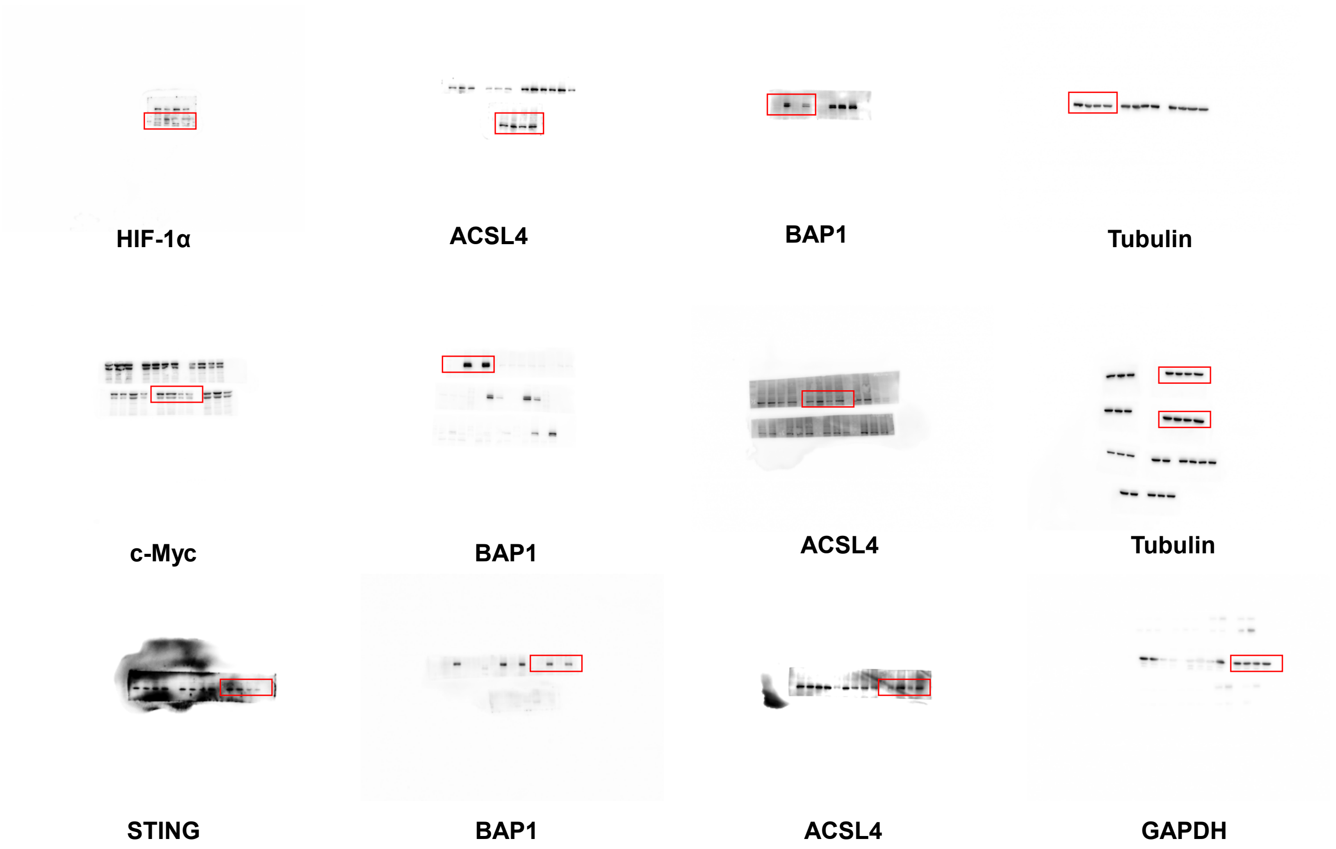

Fig 4A

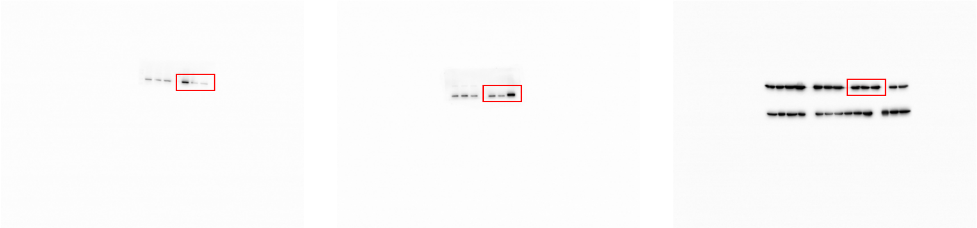

BAP1 ACSL4 Tubulin

Fig 4I

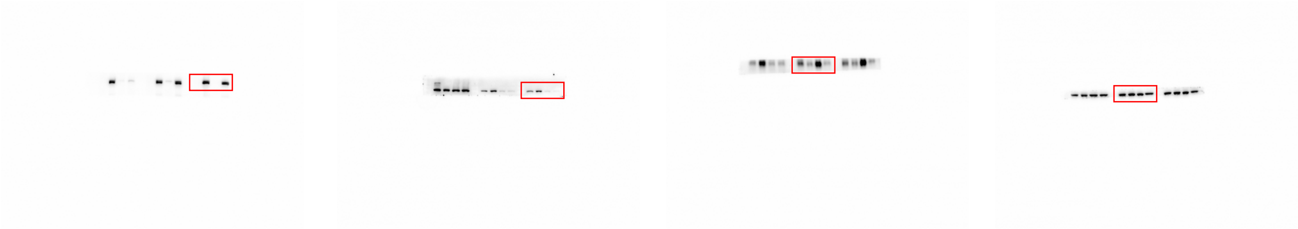

BAP1 ACSL4 SLC7A11 Tubulin

Fig S4E

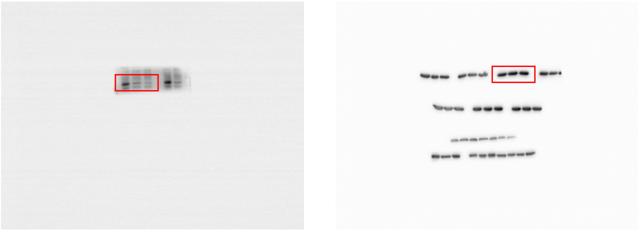

ACSL4 Tubulin

Fig 6A

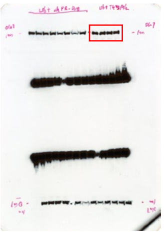
OGT-input

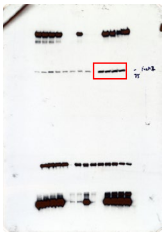
FOXK2-input

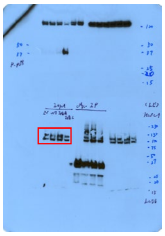
HCFC1-input

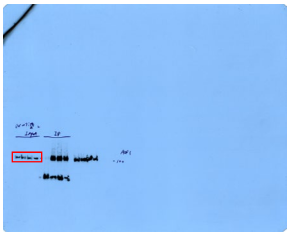
KDM1B-input

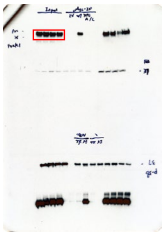
FOXK1-input

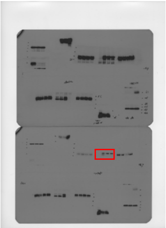
OGT-IP

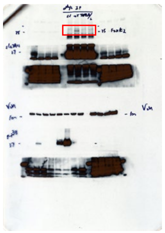
FOXK2-IP

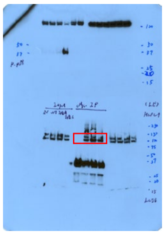
HCFC1-IP

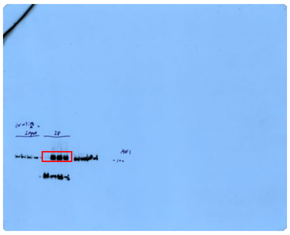
KDM1B-IP

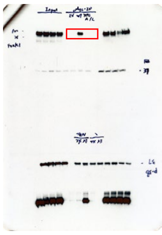
FOXK1-IP

Fig 6B

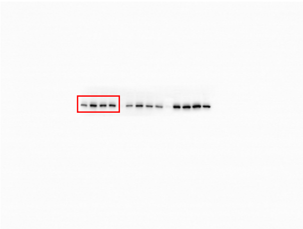
ACSL4

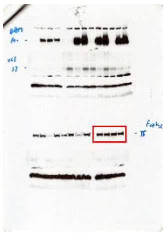
FOXK2

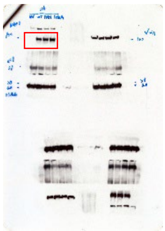
BAP1

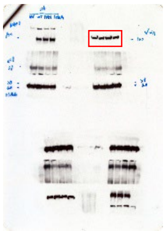
Vinculin

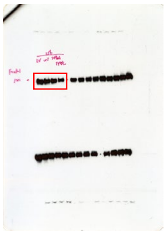
FOXK1

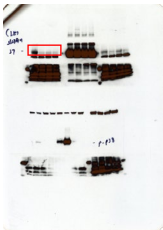
SLC7A11

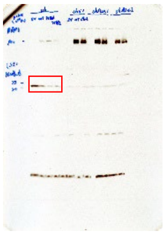
H2Aub

Fig 6C

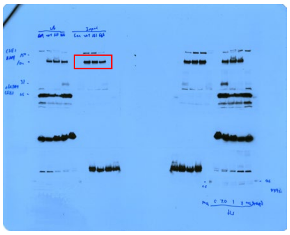
BAP1-input

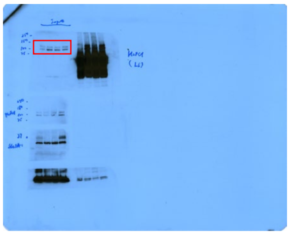
HCFC1-input

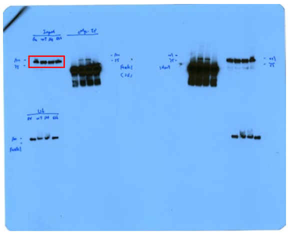
FOXK1-input

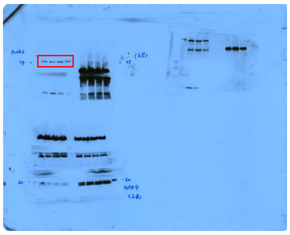
FOXK2-input

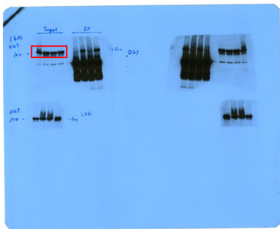
OGT-input

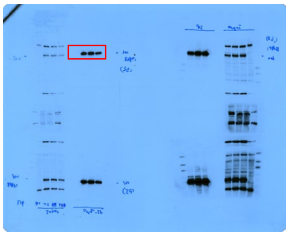
BAP1-IP

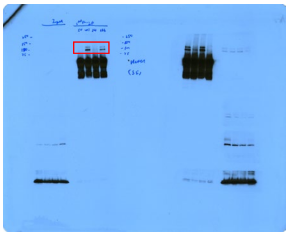
HCFC1-IP

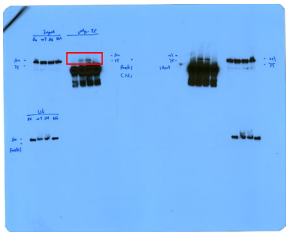
FOXK1-IP

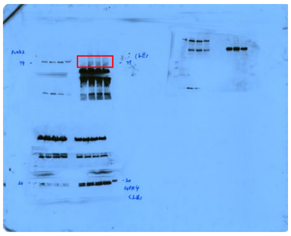
FOXK2-IP

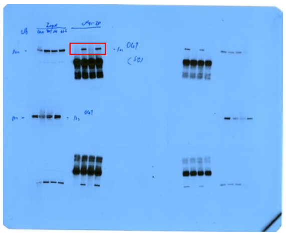
OGT-IP

Fig 6D

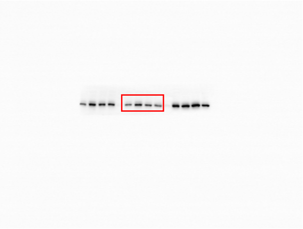
ACSL4

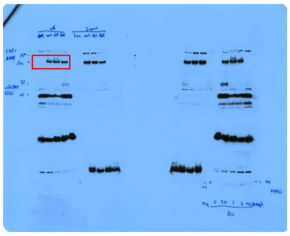
BAP1

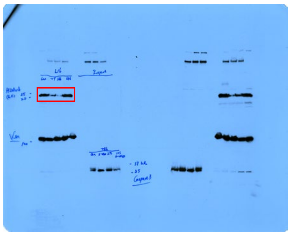
H2Aub

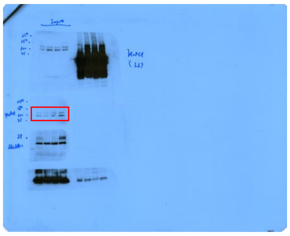
HCFC1

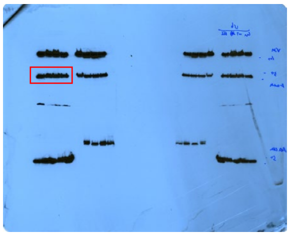
β-actin

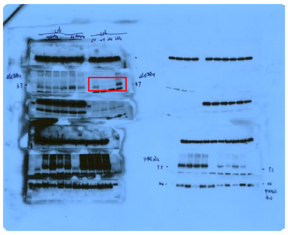
SLC7A11

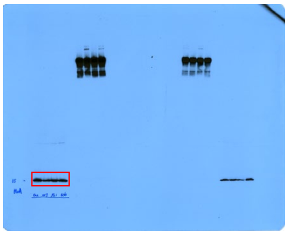
H2A

Fig 6I

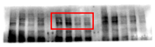
ASXL1

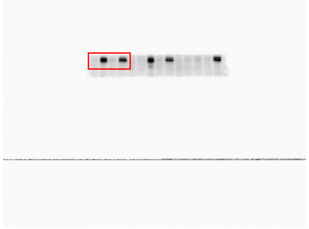
BAP1

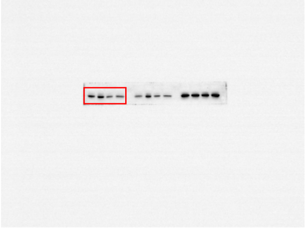
ACSL4

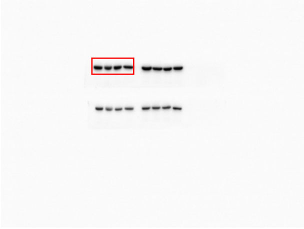
Tubulin

Fig S6A

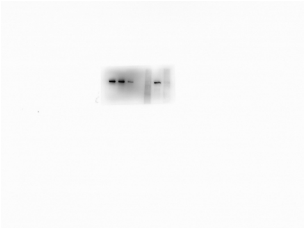
c-Myc

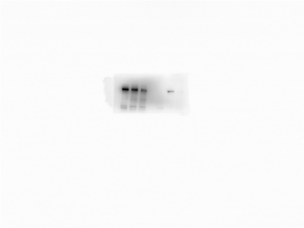
ASXL1

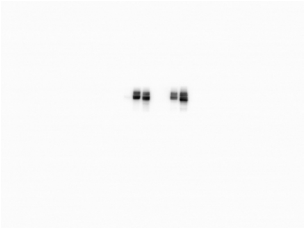
BAP1

Flag

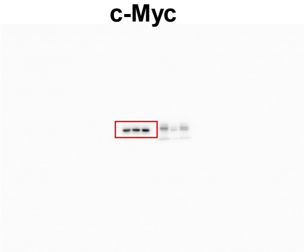
Tubulin

Fig S6B

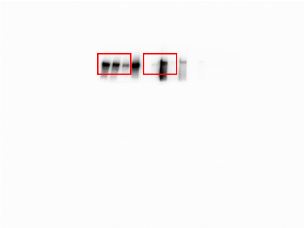
ASXL2

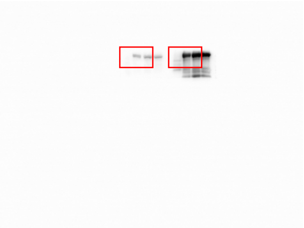
cMyc

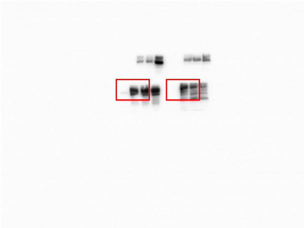
BAP1

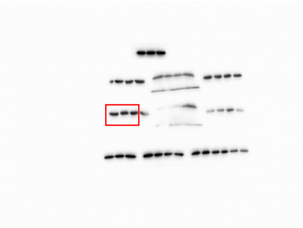
Tubulin

Fig S6G

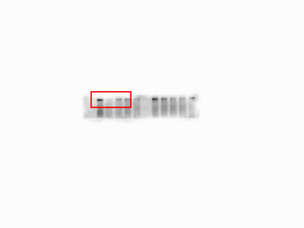
ASXL1

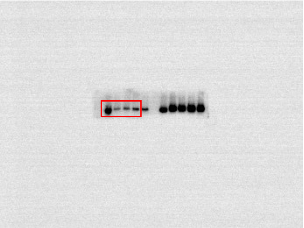
ACSL4

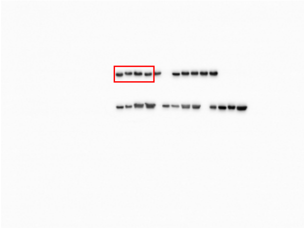
Tubulin

Fig S6H

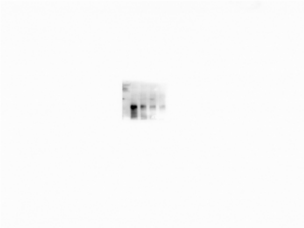
ASXL2

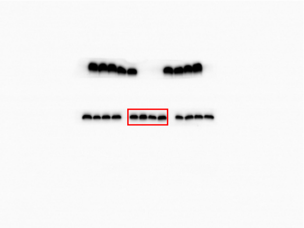
Tubulin

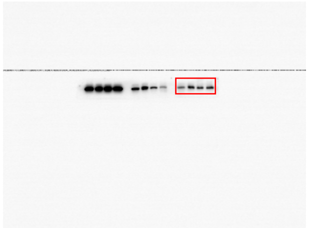
ACSL4

Fig S6L

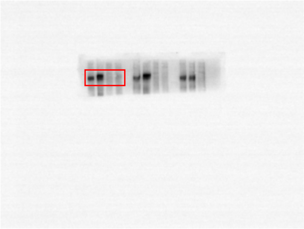
ASXL2

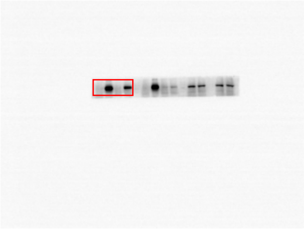
BAP1

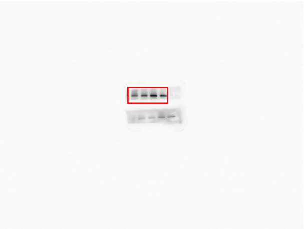
ACSL4

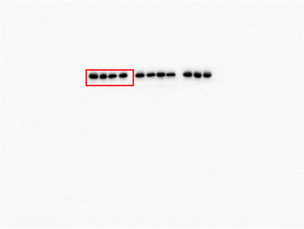
Tubulin
